# Dynamically reconfigurable topological routing in nonlinear photonic systems

**DOI:** 10.1038/s41377-025-02108-1

**Published:** 2026-01-03

**Authors:** Stephan Wong, Simon Betzold, Sven Höfling, Alexander Cerjan

**Affiliations:** 1 Center for Integrated Nanotechnologies, Sandia National Laboratories, Albuquerque, NM 87185 USA; 2 Julius-Maximilians-Universität Würzburg, Physikalisches Institut, and Würzburg-Dresden Cluster of Excellence ct.qmat, Lehrstuhl für Technische Physik, Am Hubland, Würzburg 97074 Germany

**Keywords:** Polaritons, Photonic crystals

## Abstract

The propagation path of topologically protected states is bound to the interface between regions with different topology, and as such, the functionality of linear photonic devices leveraging these states is fixed during fabrication. Here, we propose a mechanism for dynamic control over a driven dissipative system’s local topology, yielding reconfigurable topological interfaces and thus tunable paths for protected routing. We illustrate our approach in non-resonantly pumped polariton lattices, where the nonlinear interaction between the polaritons and the exciton reservoir due to non-resonant pumping can yield picosecond-scale changes in the propagation paths of the chiral edge states. To analytically confirm the numerically observed topological dynamics, we generalize the spectral localizer framework to non-linear non-Hermitian Chern materials and apply this framework to a continuous model of the polariton system based on a driven-dissipative Gross-Pitaevskii equation. In doing so, we show that the local changes in the polariton lattice’s topology are captured by a local Chern marker. Looking forward, we anticipate such reconfigurable topological routing will enable the realization of novel classes of topological photonic devices.

## Abstract (web summary)

Dynamic reconfiguration of photonic topological interfaces enables ultrafast routing at telecommunications wavelengths by leveraging nonlinear exciton–polariton interactions.

## Graphical abstract

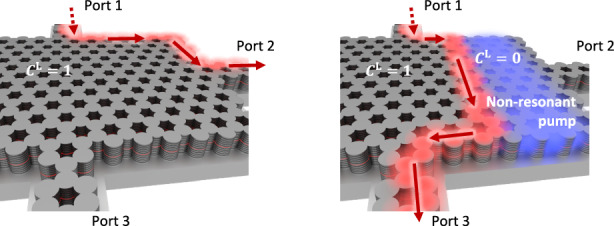

## Introduction

Over the past decade, topological photonics has emerged as a promising collection of physical principles for controlling the flow of light. For example, there is significant interest in harnessing topological phenomena for potential applications and integration into nanophotonic systems to realize robust photon routing^1–11^ and create lasers with increased coherence^12–16^. In particular, the chiral edge modes supported by photonic Chern insulators are an especially enticing class of states for designing next-generation optical devices, as these states both enable nonreciprocal transport and are robust against fabrication imperfections. Prior studies have observed chiral edge modes in a variety of photonic platforms, including photonic crystals with gyro-optical materials^17^, shifted ring-resonator arrays^18,19^, helical waveguide arrays^20^, and more recently exciton-polariton lattices^21^.

However, the interface localization of a photonic Chern heterostructure’s chiral edge modes also presents a substantial limitation on device design: these states’ propagation path is fixed by the system’s geometry and cannot be readily altered after the device is fabricated. Although there are previous proposals for reconfigurable topological systems, they are predominantly in the microwave regime with slow reconfigurability time scales in the range of milliseconds^22,23^, and are thus not technologically relevant for integrated nanotechnologies. At telecommunication wavelengths, prior work on reconfigurable photonics has either been realized through dynamically controlling each lattice element^24^, an approach that is challenging to scale, or through tunable spatially non-uniform non-Hermiticity^25^, which requires introducing large material absorptivities. As such, photonic systems rooted in linear topology are best suited to devices tailored for a single, static function, but are poor candidates for applications requiring dynamic behavior, such as routing. In addition, while many studies in nonlinear systems have considered topological solitons that can be injected at different lattice sites^26–31^, these states are still constrained to move along the lattice’s structural boundary or remain confined in its bulk^32–34^, and thus yield similar design constraints as linear systems. Altogether, an ideal reconfigurable topological platform would instead exhibit a scalable, fast, and dynamic method for changing the system’s local Chern phase without introducing additional propagation losses, so that a reconfigurable router can take full advantage of a chiral edge mode’s inherent reflectionless propagation^35^ and robustness against dephasing^36^.

**Fig. 1:**
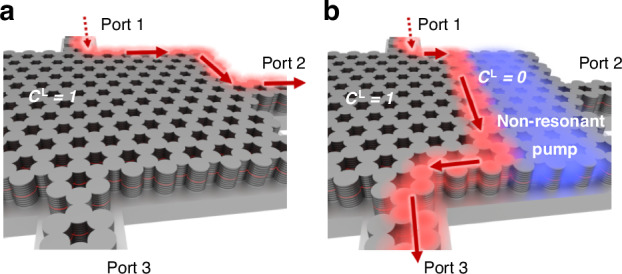
Scheme for dynamical control over the topological mode’s propagation path. **a** Schematic of an exciton-polariton topological Chern insulator. Energy is injected into the chiral edge mode (red) with a resonant laser and propagates along the boundary of the lattice to the output Port 2. **b** Illuminating the same nonlinear lattice with a non-resonant pump (blue) renders the lattice locally topologically trivial and leads to a different path of propagation for the topological mode. The exact path depends on the shape of the pump pattern. In (**b**), the topological mode propagates to the output Port 3, while avoiding the output Port 2

Here, we propose an approach for realizing fast reconfigurable local Chern topology in driven-dissipative photonic systems and numerically illustrate our method in a non-resonantly pumped exciton-polariton platform^37^ operating at telecommunication wavelengths. In particular, starting with a topologically non-trivial polariton lattice^21,37–41^ (Fig. 1a), a spatially non-uniform incident pump can locally change the system’s topology to be trivial through a nonlinear matter-matter interaction between the exciton reservoir and the polaritons. Thus, an incident signal propagating in a chiral edge state is guaranteed to follow the new topological boundary determined by the non-resonant pump pattern (Fig. 1b). As the exciton reservoir is dissipative, if the non-resonant pump is turned off, the full lattice returns to its original topological phase after a characteristic decay time, but this dissipation does not strongly influence the polaritons such that their propagation remains relatively lossless. Moreover, by superposing the reconfigurable Chern topology features using multiple non-resonant pump patterns and pump amplitudes, we realize reconfigurable multi-channel topologically protected routing via different topological interfaces in different energy ranges. To characterize the local topological dynamics we observe in our system, we develop a nonlinear non-Hermitian generalization of the spectral localizer framework^42–47^ to use with a continuum model of the exciton-polariton lattice based on the driven-dissipative Gross-Pitaevskii equations for experimentally realizable parameters^21^. Given the possible tiny extent of a pumped region, the non-linear non-Hermitian local Chern marker we establish provides a rigorous and quantitative understanding of the lattice’s local topology. Overall, while there has been growing interest in inducing topological phase changes via optical pumping without relying on external magnetic fields^37,48–56^, our results show that it is possible to dynamically reconfigure a system’s topological interfaces post-fabrication by leveraging a nonlinear response, a phenomenon that should be available in a variety of systems featuring nonlinear interactions and may be of practical use for multitasking devices while retaining the robustness of topological protected edge modes.

## Results

### Nonlinearly-induced topological interface

To illustrate dynamic control of local topology, we consider Chern polariton lattices consisting of quantum wells embedded in a honeycomb array of vertical microcavities^21,57^ under non-resonant pumping. In particular, the non-resonant pumping populates an exciton reservoir, and the dynamics of the polaritons ${\boldsymbol{\psi }}({\boldsymbol{x}},t)$ with the exciton reservoir ${n}_{r}({\boldsymbol{x}},t)$ are described by a driven-dissipative Gross–Pitaevskii equation^54,58^

1 $$i\hslash \frac{\partial }{\partial t}{\boldsymbol{\psi }}={H}_{0}{\boldsymbol{\psi }}-i\hslash \frac{{\gamma }_{c}}{2}{\boldsymbol{\psi }}+{g}_{c}{\left|{\boldsymbol{\psi }}\right|}^{2}{\boldsymbol{\psi }}+\left({g}_{r}+i\hslash \frac{R}{2}\right){n}_{r}{\boldsymbol{\psi }}+{S}_{\mathrm{probe}}$$

2 $$\frac{\partial }{\partial t}{n}_{r}=-\left({\gamma }_{r}+R{\left|{\boldsymbol{\psi }}\right|}^{2}\right){n}_{r}+{S}_{{\rm{pump}}}$$

Here, ${H}_{0}$ is the linear polariton Hamiltonian, ${\gamma }_{c}$ and ${\gamma }_{r}$ are the relaxation rates for the polariton state and exciton reservoir, ${g}_{c}$ and ${g}_{r}$ are the polariton-polariton and polariton-exciton interaction strengths, $R$ is the amplification rate of the polariton state due to stimulated scattering from the reservoir, ${S}_{{\rm{probe}}}({\boldsymbol{x}},t)$ is the resonant probe for directly exciting the polaritons, and ${S}_{{\rm{pump}}}({\boldsymbol{x}},t)$ is the non-resonant pump used for injecting free carriers. When ${S}_{{\rm{pump}}}\left({\boldsymbol{x}},t\right)=0$, the non-trivial topology in the polariton lattice^21,37–41^ results from the interplay of an external magnetic field and an effective spin-orbit coupling. The external magnetic field breaks time-reversal symmetry, and induces a Zeeman splitting between the spin-up and spin-down excitons, while the effective spin-orbit coupling originating from the coupling between the transverse electric (TE) and magnetic (TM) photonic modes opens a topological gap by coupling the two spin sectors. The corresponding Hamiltonian *H*_0_ is given, in the polariton spin basis ${\boldsymbol{\psi }}\left({\boldsymbol{x}}\right)=\left[{{\boldsymbol{\psi }}}_{+}\left({\boldsymbol{x}}\right),{{\boldsymbol{\psi }}}_{-}\left({\boldsymbol{x}}\right)\right]$, by

3 $${H}_{0}=\left(\begin{array}{cc}-\frac{\hslash }{2m}{\nabla }^{2}+V\left({\boldsymbol{x}}\right)+\frac{1}{2}{\Delta }_{{\rm{eff}}} & -{\beta }_{{\rm{eff}}}{\left({\partial }_{x}-i{\partial }_{y}\right)}^{2}\\ -{\beta }_{{\rm{eff}}}{\left({\partial }_{x}+i{\partial }_{y}\right)}^{2} & -\frac{\hslash }{2m}{\nabla }^{2}+V\left({\boldsymbol{x}}\right)-\frac{1}{2}{\Delta }_{{\rm{eff}}}\end{array}\right)$$

where $m$ is the polariton mass, $V\left({\boldsymbol{x}}\right)$ is the polariton potential patterned as a honeycomb lattice, ${\beta }_{{\rm{eff}}}$ is the spin-orbit coupling strength, and ${\Delta }_{{\rm{eff}}}$ is the Zeeman coefficient. When ${S}_{{\rm{pump}}}\left({\boldsymbol{x}},t\right)\ne 0$, it acts as an external dynamical perturbation to locally blueshift the band structure through the creation of the steady-state exciton reservoir ${n}_{r}({\boldsymbol{x}},t)$. Altogether, the instantaneous polariton Hamiltonian $H\left(t,{\boldsymbol{\psi }},{n}_{r}\right)$ is thus non-Hermitian and nonlinear,

4 $$H\left(t,{\boldsymbol{\psi }},{n}_{r}\right)={H}_{0}+i\Gamma \left({\boldsymbol{\psi }},{n}_{r}\right)+B\left({\boldsymbol{\psi }},{n}_{r}\right)$$

where $i\Gamma \left({\boldsymbol{\psi }},{n}_{r}\right)=-\hslash \frac{{\gamma }_{c}}{2}+\hslash \frac{R}{2}{n}_{r}$ gathers the non-Hermitian terms in Eq. (1) from the driven-dissipative system, and $B\left({\boldsymbol{\psi }},{n}_{r}\right)={g}_{c}{\left|{\boldsymbol{\psi }}\right|}^{2}+{g}_{r}{n}_{r}$ is the blueshift arising from the repulsive nonlinear interaction between the polaritons and the exciton reservoir^58–62^.

To identify the polariton system’s topological dynamics^63,64^ directly from the continuous Hamiltonian model [Eq. (4)], we generalize the two-dimensional (2D) spectral localizer for static non-Hermitian systems^45,46^ to incorporate nonlinearities. In particular, the polariton lattice’s topology can be classified at a specified location and energy with a given occupation using the instantaneous nonlinear non-Hermitian spectral localizer ${L}_{\left({\boldsymbol{x}},E\right)}$,

5 $${L}_{\left({\boldsymbol{x}},E\right)}\left(X,Y,H\left(t,{\boldsymbol{\psi }},{n}_{r}\right)\right)=\left(\begin{array}{cc}\left[H\left(t,{\boldsymbol{\psi }},{n}_{r}\right)-E{\bf{1}}\right] & \kappa \left(X-x{\bf{1}}\right)-i\kappa \left(Y-y{\bf{1}}\right)\\ \kappa \left(X-x{\bf{1}}\right)+i\kappa \left(Y-y{\bf{1}}\right) & -{\left[H\left(t,{\boldsymbol{\psi }},{n}_{r}\right)-E{\bf{1}}\right]}^{\dagger }\end{array}\right)$$

where *H* is the Hamiltonian matrix derived from the finite-difference discretization of the instantaneous continuous model [Eq. (4)], *X* and *Y* are the position matrices that (in this basis) are diagonal with entries corresponding to the real-space coordinates of the finite-difference degrees-of-freedom in the *x*- and *y*-directions, and **1** is the identity matrix. In Eq. (5), the subscript $({\boldsymbol{x}},E)$ indicates the location (${\boldsymbol{x}}$) and energy ($E$) where the local topology will be classified, and $\kappa$ is a scaling coefficient that enforces consistent units between the position and Hamiltonian matrices. $\kappa$ also ensures balanced spectral weights on the system’s position information relative to its Hamiltonian, and is typically of the order $\kappa \sim {E}_{{\rm{gap}}}/L$^44,46,65^ where ${E}_{{\rm{gap}}}$ is the relevant spectral gap’s width and *L* the length of the finite system considered. Using ${L}_{\left({\boldsymbol{x}},E\right)}$, instantaneous local topology at some spatial-energy coordinate $({\boldsymbol{x}},E)$ can be determined using the local Chern number ${C}_{\left({\boldsymbol{x}},E\right)}^{{\rm{L}}}$^42,45^

6 $${C}_{\left({\boldsymbol{x}},E\right)}^{{\rm{L}}}\left(X,Y,H\left(t,{\boldsymbol{\psi }},{n}_{r}\right)\right)=\frac{1}{2}{\rm{si}}{{\rm{g}}}_{{\mathbb{R}}}\left[{L}_{\left({\boldsymbol{x}},E\right)}\left(X,Y,H\left(t,{\boldsymbol{\psi }},{n}_{r}\right)\right)\right]$$

where ${\rm{si}}{{\rm{g}}}_{{\mathbb{R}}}\left[M\right]$ is the signature of the line-gapped matrix *M*, i.e., the difference between the number of eigenvalues with positive and negative real parts. Note, ${C}_{\left({\boldsymbol{x}},E\right)}^{{\rm{L}}}$ is provably equal to the global Chern number for lossless crystalline gapped systems with *E* chosen in the relevant band gap^44^. Altogether, the topology of the exciton-polariton lattice can be identified using the local Chern number ${C}_{\left({{\boldsymbol{x}}}_{{0}},E\right)}^{{\rm{L}}}$ with ${{\boldsymbol{x}}}_{{0}}$ chosen inside the system’s bulk and *E* the given energy of interest [see Supplementary Note 1 for further details].

**Fig. 2:**
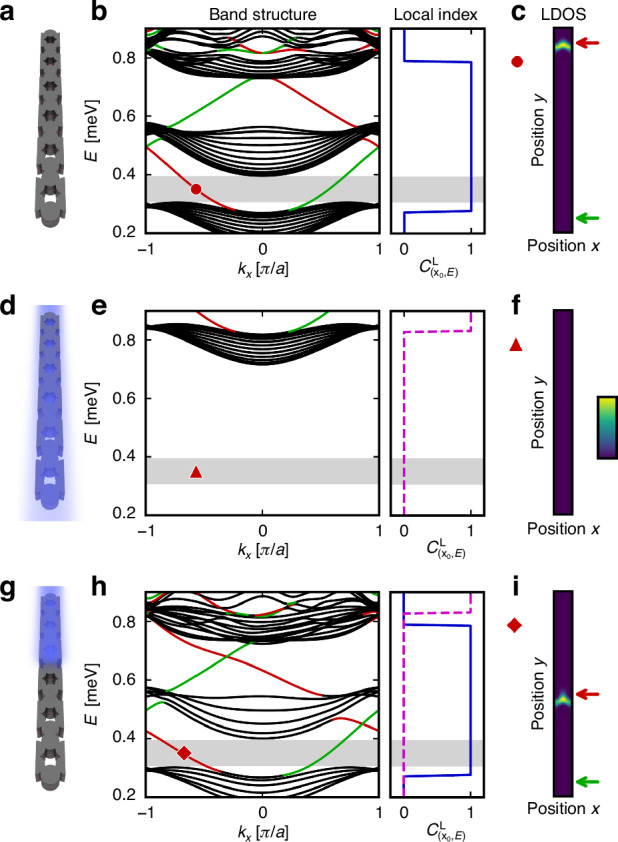
Change of the topology due to reservoir-induced blueshift. **a** Schematic of the potential landscape of the ribbon polariton lattice arranged in a honeycomb lattice. **b** Ribbon band structure and corresponding local Chern number ${C}_{\left({{\boldsymbol{x}}}_{{0}},E\right)}^{{\rm{L}}}$ for ${{\boldsymbol{x}}}_{{0}}$ in the system’s center. In the band structure, the black lines correspond to bulk modes, and the green (red) lines denote the chiral edge mode dispersion localized at the bottom (top) side of the lattice, as shown by the color-coded arrows in (**b**). The gray shaded area indicates the energy range of interest. **c** Local density of states (LDOS) of the red line at *E* = 0.35 meV, ${k}_{x}=-0.6\left[\pi /a\right]$. **d**–**f** Same as (**a**)–(**c**) but with a blue overlay depicting the blueshift of the whole ribbon structure. **g**–**i** Same as (**a**)–(**c**) but with the blueshift applied only to half of the ribbon lattice. The red (and green) lines in the band structure correspond to the topological edge modes localized at the interface between the blueshifted and non-blueshifted areas (and at the bottom edge of the lattice). In (**h**), the local Chern number obtained from both the pumped (dashed magenta line) and unpumped (solid blue line) regions are shown. **i** LDOS of the red line at *E* = 0.35 meV, ${k}_{x}=-0.7\left[\pi /a\right]$. Simulations use a reservoir-induced blueshift of ${E}_{{\rm{blueshift}}}=0.55\,{\rm{meV}}$ and spectral localizer calculations use *κ* = 0.015 meV µm^−1^ and a finite 2D system of size 26.1 µm × 23.7 µm

The nonlinearity inherent in the exciton–polariton system can yield a shift in the lattice’s local topology. In particular, we consider a ribbon geometry of the 2D system, i.e., periodic in one direction and finite in the other [Fig. 2a], and use experimentally realizable parameters from ref. ^21^. The ribbon band structure of the unpumped system [Fig. 2b] features a topologically non-trivial band gap [gray shaded area] with corresponding chiral edge modes [see red/green solid lines]. The right panel of Fig. 2b indicates the local Chern number as a function of energy, confirming the unpumped system’s topology. As the entire lattice is non-trivial, there is a topological interface at the lattice’s boundaries and therefore the chiral edge modes can be identified in the local density of states (LDOS) at the structure’s edges [Fig. 2c].

However, by including a reservoir-induced blueshift, the topology at the given energy range of interest can change. For example, by non-resonantly pumping on the entire lattice, the polariton potential landscape blueshifts [Fig. 2d] and thus the system’s band structure does as well [Fig. 2e]. As such, this blueshift induces the lattice’s topology to become trivial in the chosen energy range [gray shaded area in Fig. 2e]. Therefore, if only half of the lattice is non-resonantly pumped [Fig. 2g, h], only that portion of the lattice will be rendered trivial in the energy range of interest. In other words, such non-uniform pumping creates a topological interface between the pumped and unpumped regions within the lattice. Moreover, the corresponding ribbon band structure for this nonlinearly induced “heterostructure” reveals the existence of chiral edge modes that cross the bulk band gap at the chosen energy [Fig. 2h] that are associated to states localized at the newly formed topological interface [Fig. 2i] and at the lattice’s boundary. Notably, the topological transition across the “heterostructure” interface is attributed to a reservoir-induced blueshift, where instead of changing the overall topology of the exciton-polariton manifold, the repulsive nonlinear interaction between the exciton reservoir and the exciton-polariton will shift the energies of the polariton states relative to the unpumped regions.

### Topological routing with non-resonant pump patterns

**Fig. 3:**
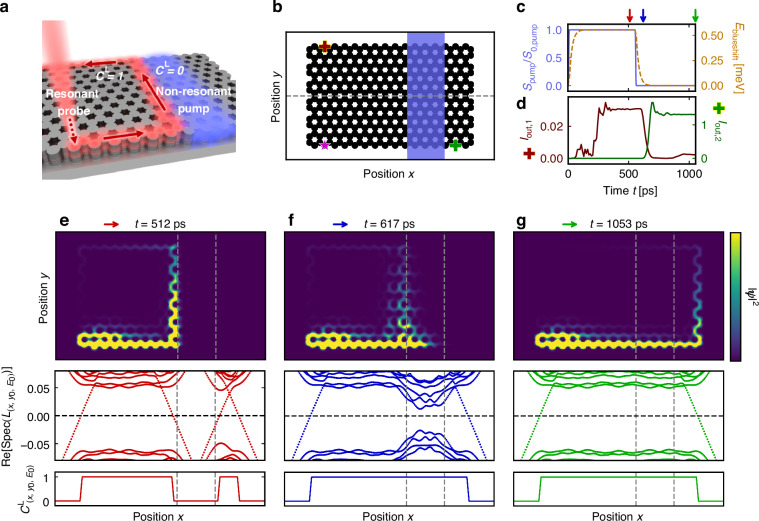
Reconfigurable topological routing with non-resonant pumping. **a** Schematic of the polariton lattice where the excited topological edge mode’s propagation path (red) has been re-routed by non-resonant illumination (blue). **b** Potential landscape $V(x,y)$ of the polariton honeycomb lattice. The black and white regions correspond to potentials of *V* = 6 meV and *V* = 0 meV, respectively. The blue shaded area depicts the pump pattern and the magenta star indicates the position of the probe source. **c** Temporal evolution of the non-resonant pump power (blue solid line) and the induced blueshift (orange dashed line). **d** Output intensity integrated over a width of 2 µm in the *y*-direction, at the positions given by the dark red and dark green crosses in (**b**). **e**–**g** Snapshot of the total intensity of the polariton ${\left|{\boldsymbol{\psi }}\right|}^{2}={\left|{{\boldsymbol{\psi }}}_{+}\right|}^{2}+{\left|{{\boldsymbol{\psi }}}_{-}\right|}^{2}$ at the time indicated by the color-coded arrows in (**c**), with the corresponding eigenvalues of the spectral localizer $\mathrm{Re}\left[{\rm{Spec}}\left({L}_{(x,{y}_{0},{E}_{0})}\right)\right]$ and the local Chern number ${C}_{\left(x,{y}_{0},{E}_{0}\right)}^{{\rm{L}}}$ along the gray dashed line in (**b**) and at *E*_0_ = 0.35 meV. The intensity plots use the same color scale, and the gray dashed lines indicate the non-resonant pump pattern. The parameter values for the Hamiltonian are the same as in Fig. 2, while the pump amplitude ${S}_{0,{\rm{pump}}}=2.5$ ps^−1^ µm^−2^, probe amplitude ${S}_{0,{\rm{probe}}}=0.5$ ps^−1^ µm^−2^, resonant frequency $\hslash {\omega }_{s}=0.35\,{\rm{meV}}$ [see Methods], and *κ* = 0.015 meVµm^−1^

A prototypical system demonstrating a reconfigurable Chern interface in a polariton lattice is shown in Fig. 3a, b. Here, a non-resonant pump with amplitude below the condensate threshold [blue shaded area] is used to populate an exciton reservoir that reaches a steady-state due to its inherent dissipation, while a resonant probe source [magenta star] excites the chiral edge modes. As such, after the exciton reservoir becomes sufficiently populated, the polaritons experience a blueshift due to the repulsive nonlinear interactions according to Eqs. (1)–(2), locally modifying the polariton’s topology, and creating new topological interfaces that were not present in the unpumped system. Once the non-resonant pump is turned off, the exciton reservoir’s population dissipates, returning the polariton’s local topology to its original configuration. Moreover, we emphasize that the resonant probe ${S}_{{\rm{probe}}}({\boldsymbol{x}},t)$ is a weak coherent excitation introduced to launch the topological edge modes. As this probe operates below the condensation threshold and does not significantly populate the exciton reservoir (because the topological edge modes are resonantly excited), the linewidth of the excited topological exciton-polaritons is not subject to the same broadening mechanism as the reservoir-coupled dynamics. As such, the linewidth values used in our simulations refer to this resonant signal.

The complete temporal evolution of our non-resonantly pumped exciton-polariton lattice is shown in Fig. 3c, g, where we consider a system that is initially pumped in one region, but that pump is later turned off. The dynamics of the local blueshift ${E}_{{\rm{blueshift}}}\left(t\right)={g}_{r}{n}_{r}(t)$ in the pumped region are shown in Fig. 3c, which demonstrates that the population of the exciton reservoir initially saturates at a steady-state (with ${E}_{{\rm{blueshift}}}=0.55\,{\rm{meV}}$) after a characteristic time given by ${\gamma }_{r}$ before becoming completely depleted once the pump amplitude is switched off. Moreover, throughout the evolution of the reservoir population, real-space snapshots of the topological dynamics of this non-Hermitian and non-linear system can be found using the spectral localizer framework, as shown in the bottom panels of Fig. 3e–g. Note, given the tiny extent of the pumped region, topological band theory would only provide qualitative insights of the topology of the system, which may break down for this region’s size. Instead, by monitoring the real parts of the spectral localizer’s eigenvalues ${\mathrm{Re}}\left[{\rm{Spec}}\left({L}_{(x,{y}_{0},{E}_{0})}\right)\right]$ for choices of *x* along the gray dashed line in Fig. 3b and at the topological mode’s expected energy *E*_0_ = 0.35 meV [see Fig. 2], the change in the polariton’s topology can be directly observed in a quantitative and rigorous manner, as this spectral flow (at a given *t*) is responsible for local shifts in ${C}_{\left(x,{y}_{0},{E}_{0}\right)}^{{\rm{L}}}$. At the outer edge of the lattice, one of the eigenvalues of the spectral localizer crosses zero and the local Chern number changes from ${C}_{\left(x,{y}_{0},{E}_{0}\right)}^{{\rm{L}}}=0\to 1$ (as $x$ varies from outside the lattice to inside) because the lattice is topologically non-trivial while the surrounding empty space is trivial. In the presence of the populated exciton reservoir, there is an additional change in the polariton’s topology from the shape of the non-resonant pump: the local Chern number is trivial ${C}_{\left({\boldsymbol{x}},E\right)}^{{\rm{L}}}=0$ inside the pumped region [see bottom panels of Fig. 3e]. Once the pump is turned off [Fig. 3f, g], the polariton’s nonlinear interactions with the exciton reservoir dissipate and the blueshift eventually becomes negligible, such that the previously pumped region becomes topological again, removing the in-lattice topological boundary [see bottom panels of Fig. 3f, g]. Note that during this change in the exciton reservoir, the varying nonlinearly-induced blueshift may cause frequency shifts or chirps as the wave packet propagates along the newly formed interface, but any such frequency components ultimately decay due to the system’s dissipative nature.

Overall, the picosecond time-scale reconfigurability of the exciton-polariton lattice’s topology is realized thanks to the driven-dissipative nature of the system, and is particularly effective because the dominant portion of the system’s intrinsic dissipation ${\gamma }_{r}$ manifests in a different sector than the system’s propagating states such that the polariton transport is not strongly influenced. While an intrinsic loss ${\gamma }_{c}$ is present for the polariton state, this loss is only included to model realistic exciton-polariton systems and is by no means necessary to realize fast reconfigurable Chern topology [see Supplementary Note 2 for further details]. Specifically, the dynamics of the excited polariton states, shown with the real-space snapshots in Fig. 3e–g, can be summarized as follows: When the lattice is (locally) pumped, the excited polariton state at the bottom-left side of the lattice initially propagates along the edge of the topologically non-trivial lattice, before turning upward along the nonlinearly induced topological interface without being back-reflected [Fig. 3e], as the pumped region is a trivial insulator at the excited polariton energy *E* = 0.35 meV. However, when the non-resonant pump is turned off, the exciton reservoir dissipates and the previously pumped region returns to being topological. As such, there is no longer a topological interface in the system’s bulk, and the topological edge mode that was previously propagating in the bulk starts to decay as the system’s bulk becomes gapped again [Fig. 3f]. Subsequently, the excited polariton propagates along the lattice edge without being back-reflected [Fig. 3g].

The dynamics of the chiral edge mode’s propagation can be quantified by looking at the intensity at different output locations, as shown in Fig. 3d. The output intensity at the lattice’s upper left corner ${I}_{{\rm{out}},1}$ [dark red cross] reaches a plateau when the lattice is non-resonantly pumped, while the output intensity at the bottom right corner ${I}_{{\rm{out}},2}$ [dark green cross] reaches a plateau when the pump is turned off. Notably, ${I}_{{\rm{out}},2}$ reaches a steady state in approximately 70 ps with a high on/off state ratio, making it a promising candidate for ultrafast optical-switching^66^. Altogether, with the base energy of the exciton-polariton being about 1 eV^21,37–41^ [see Methods], fast reconfigurable topologically protected routing at telecommunications wavelengths is achieved solely from the dynamical control over the shape and existence of a non-resonant pump applied to the non-trivial polariton lattice, which can be realized using spatial light modulators or electrical pumping^67,68^ [see also Supplementary Note 6 for further details].

### Multi-channel topological routing with multiple non-resonant pump patterns

Beyond reconfigurable single-channel routing demonstrated in Fig. 3, the use of multiple pump patterns in the system enables reconfigurable multi-channel topological routing. While a straightforward extension of the previous results provides a recipe to simultaneously guide information along topological interface modes from different input channels to different output channels at a single frequency, the use of multiple pump patterns with different amplitudes also enables frequency-dependent reconfigurable topological routing where the propagation path of the topological edge states varies based on their frequency.

**Fig. 4:**
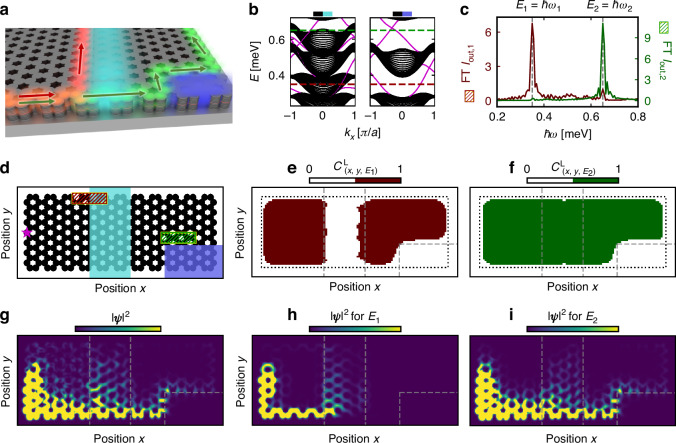
Multi-channel topological routing with non-resonant pumpings. **a** Schematic of the polariton lattice where the propagation path of excited topological edge modes (red, green) will only be re-routed either by a smaller amplitude non-resonant pump (cyan) or by a larger amplitude non-resonant pump (blue). **b** Ribbon band structures at the interface between the unpumped and pumped regions. The left band structure is for the smaller amplitude pump and corresponds to a blueshift of ${E}_{{\rm{blueshift}}}=0.11\,{\rm{meV}}$, while the larger amplitude pump corresponds to ${E}_{{\rm{blueshift}}}=0.6\,{\rm{meV}}$. In the band structure, the black lines correspond to bulk modes and the magenta lines denote the chiral edge mode dispersion localized at the interface. The red and green horizontal dashed lines denote the resonant frequencies used. **c** Fourier transform (FT) of the polariton signal over the red and green hatched areas in (**d**). **d** Potential landscape $V(x,y)$ of the polariton honeycomb lattice. The hatched regions denote where the smaller ${S}_{0,{\rm{pump}}}^{(0)}$ (cyan) and larger ${S}_{0,{\rm{pump}}}^{(1)}$ (blue) pump amplitudes are applied. The magenta star indicates the position of the probe source. **e**, **f** Local Chern numbers ${C}_{\left(x,y,{E}_{i}\right)}^{{\rm{L}}}$ calculated over the system at the energy *E*_*i*_ = *E*_0_ = 0.35 meV and *E*_*i*_ = *E*_0_ = 0.65 meV, respectively. **g** Snapshot of the total intensity of the polariton ${\left|{\boldsymbol{\psi }}\right|}^{2}={\left|{{\boldsymbol{\psi }}}_{+}\right|}^{2}+{\left|{{\boldsymbol{\psi }}}_{-}\right|}^{2}$. **h**, **i** Snapshot of the total intensity of the polariton |*ψ* | ^2^ when the probe source is resonantly exciting only either at the ${E}_{1}=\hslash {\omega }_{s,1}$ or ${E}_{2}=\hslash {\omega }_{s,2}$ energy, respectively. The intensity plots in (**g**)–(**i**) use the same color scale. In (**e**)–(**i**), the snapshot of the topological dynamics has been taken at *t* = 658 ps, and the gray dashed lines indicate the non-resonant pump patterns. The black dotted lines in (**e**), (**f**) indicate the lattice boundary. The parameter values for the Hamiltonian are the same as in Fig. 2, the smaller pump amplitude ${S}_{0,{\rm{pump}}}^{(0)}$ = 0.5 ps^−1^ µm^−2^, larger pump amplitude ${S}_{0,{\rm{pump}}}^{(1)}=2.7$ ps^−1^ µm^−2^, probe amplitude ${S}_{0,{\rm{probe}}}=0.5$ ps^−1^ µm^−2^, the resonant frequencies are $\hslash {\omega }_{s,1}=0.35\,{\rm{meV}}$ and $\hslash {\omega }_{s,2}=0.65\,{\rm{meV}}$ [see Methods], and *κ* = 0.015 meV µm^−1^

To illustrate reconfigurable multi-channel topological routing, we consider a prototypical exciton-polariton lattice where two non-resonant pump patterns with different pump amplitudes are utilized, as shown in Fig. 4a, d, while a single probe source is used to excite topological edge modes at several energies. In particular, one pump pattern has a pump amplitude small enough to only induce a change of the system topology in a lower energy range, while the second pump is strong enough to modify the system’s topology in a higher energy range. Altogether, the different pump amplitudes induce local blueshifts of different strengths, resulting in energy-dependent modifications to the system’s local topology and thus nonlinearly induced topological interfaces that are distinct for inputs at different energies.

A real-space snapshot of the exciton-polariton lattice’s topological dynamics is shown in Fig. 4e–g, where the nonlinearly induced blueshifts of the non-resonant pumps in the middle [cyan] and lower right corner [blue] are respectively ${E}_{{\rm{blueshift}}}=0.11\,{\rm{meV}}$ and ${E}_{{\rm{blueshift}}}=0.6\,{\rm{meV}}$ once the corresponding population of the exciton reservoir reached a steady state. As such, the local blueshift in the lattice’s middle leads to a gapless “heterostructure” interface configuration [Fig. 4b] which is topological for the lower band gap energy range while being trivial for the higher band gap energy range [see Figs. S4, S5 in Supplementary Note 4 for further details]. In contrast, the local blueshift in the lower right corner is similar to Fig. 2 and Fig. 3 [see Fig. 4b], where there is a shared bulk spectral gap at the higher energy polariton’s frequency on both sides of the nonlinearly induced topological interface. Thus, the topologically distinct domains of the system at energies in the lower and higher energy ranges are different, as plotted in Fig. 4e, f. Note that this multi-routing configuration makes use of a particular feature of the spectral localizer framework to be able to predict the existence of topological modes even in the absence of a bulk band gap^46,47,69^, such as for the gapless “heterostructure” interface between the unpumped lattice and the region with a smaller pump amplitude [cyan]. As a result, the chiral edge modes along such interfaces form “topological edge resonances” as they energetically overlap with bulk modes, although at different wave-vectors. As such, these topological edge resonances scatter into the bulk modes whenever these edge modes encounter a scatterer, such as a corner or defect, that enables wave-vector matching with the bulk modes through broken translational symmetry along the edge. Despite leaking into the bulk, these topological edge resonances are expected to exhibit robustness guaranteeing their existence similar to topological edge modes obtained from a gapped “heterostructure” interface [see Fig S6 in Supplementary Note 4 for further details]. The effect of disorder on these topological edge mode resonances results in an increased effective intrinsic loss due to increasing leakage to the bulk modes.

Overall, an excitation at *E*_1_ in the lower energy range propagates along the topological interface induced by the cyan non-resonant pump [Fig. 4h], while an excitation at *E*_2_ in the higher energy range propagates through the cyan pump area while still experiencing the topological interface formed by the blue shaded area [Fig. 4i]. Therefore, for a point source resonantly exciting topological modes at energies *E*_1_ and *E*_2_, the two topological modes will be guided to different output channels [Fig. 4g]; taking the Fourier transform (FT) of the polariton time-evolution summed over the different output-channel areas [see Fig. 4d] leads to the respective peaks at energies *E*_1_ and *E*_2_ [Fig. 4c]. These peaks in the transmission spectrum are protected against disorder, illustrating the robustness of the multichannel topological routing despite the gaplessness of one of the nonlinearly induced topological “heterostructures” [see Supplementary Note 5 for further details]. Altogether, our results demonstrate multi-channel (frequency-dependent) topological routing that can be dynamically controlled by multiple non-resonant pumps.

## Discussion

To summarize, we have presented a technologically realistic proposal for dynamic reconfigurable topological routing in nonlinear driven-dissipative photonic systems in a single device, and shown that this approach works at telecommunication wavelengths and at picosecond time scales. This method has been illustrated with non-resonantly pumped exciton-polariton lattices, and in particular, using a continuum model of the driven-dissipative Gross-Pitaevskii equations parameterized with prior experimental results^21^. Moreover, we have demonstrated dynamic control over the propagation path of the system’s chiral edge states—including its frequency-dependent behavior—through a nonlinearly induced topological phase transition stemming from spatially non-uniform non-resonant pumping of the exciton reservoir. To rigorously prove the system’s local topological phase change, we have generalized the spectral localizer framework to accommodate nonlinear non-Hermitian systems, and found a broad range of pump powers and polarizations are suitable for inducing a local nonlinear topological transition in exciton-polariton lattices. More broadly, our framework enables a rigorous and quantitative probing of the dynamical change of the topology over time and within regions containing very few unit cells where topological band theory would be unreliable. As such, we anticipate the spectral localizer to prove valuable for characterizing topological mechanisms unique to nonlinear, non-Hermitian systems.

Note that while the non-resonant pump on the exciton-reservoir can effectively broaden the linewidth of the blueshifted bands, we do not expect this enhanced linewidth to substantially influence our results. Considering a greater linewidth (for example 0.1 meV^21,70^) only in the pumped region due to the reservoir, the reconfigurable topological routing system discussed here (Figs. 2, 3) would still have a topological band gap at the probe’s frequency. In the case where the heterostructure lacks a bulk spectral gap (Fig. 4), the spectral localizer is still able to classify the system’s local topology^46,69^, and thus the increased linewidth will not affect the identification of nonlinearly induced topological boundaries. While it is possible for the exciton-reservoir to populate the polariton state in the gapless heterostructure as there are bulk reservoir states with the same energy as the polariton probe, increasing the measured probe’s linewidth, this coupling will be suppressed as these bulk modes are not momentum-matched to the polariton edge mode and the non-resonant pump generally conserves the system’s translational symmetry along the induced topological boundary. Moreover, one could circumvent many of these possible challenges by using alternative tunable methods for obtaining blueshifts in the polariton bands, such as the Stark effect via electrical gating^71–74^, which enables such blueshifting without creating a broadened exciton reservoir, thereby resulting in much narrower effective linewidths. Similarly, one could also optimize the geometry of the lattice or the orbitals used to have a better bulk band dispersion to accommodate the broadened linewidth.

Overall, compared with previous proposals operating at the telecommunication regime^24,25^, our approach represents a significant technological advancement, avoiding large losses that hinder topological transport and overcoming the limitations of unscalable multidimensional dynamic control over each element in a system, and uniquely enables reconfigurable multi-channel topological routing, as opposed to single channel routing. Additionally, while reconfigurable topology has been realized one-dimensional (1D) systems^32–34,74^, these 1D topological systems only protect stationary states, and fundamentally differ from the propagating edge states in 2D Chern insulators. Looking forward, the possibility of realizing reconfigurable topological Chern photonic devices provides a new route to achieving reflectionless non-reciprocal routing, a capability with applications in a variety of communication technologies, while also enabling the exploration of topological aspects in dynamically modulated photonic structures^75–77^. Furthermore, as dynamical tuning of a system’s local topology is rooted in external control of a system’s nonlinear interactions, we expect nonlinearly induced topological phase transitions can be achieved in platforms featuring multiwave mixing^78–81^ through a second illumination source or perturbation, or in other strongly coupled composite systems such as phonons-polariton lattices with induced phonon-phonon interactions^82^.

## Methods

### Numerical methods and parameter values

The polariton lattice is a honeycomb lattice with lattice constant *a* = 2.95 µm (center-to-center distance is 1.7 µm), and rod radius 1 µm. The parameters of the Hamiltonian are chosen based on Ref^21^. where the polariton mass is *m* = 1.3 × 10^−4^*m*_0_ with ${m}_{0}$ the free electron mass, the spin-orbit coupling strength is ${\beta }_{{\rm{eff}}}=0.2$ meVµm^2^ and the Zeeman coefficient is ${\Delta }_{{\rm{eff}}}=-0.3\,{\rm{meV}}$. Without loss of generality, the bare energy of the exciton-polaritons ${\widetilde{E}}_{0}\, \sim \,1\,{\rm{eV}}$^21,37–41^ has not been taken into account in the Hamiltonian, as ${\widetilde{E}}_{0}$ only accounts for an energy shift of all the bands. The ribbon band structure is calculated from the ribbon geometry with periodic boundary conditions on the left and right boundaries, and Dirichlet boundary conditions on the top and bottom boundaries.

To integrate the rate equations given by the driven-dissipative Gross-Pitaevskii equations [Eqs. (1)–(2)], the time *t* is re-scaled to ${t}^{{\prime} }=t/\hslash$ and a third-order Adams-Bashforth method is utilized with a time step $d{t}^{{\prime} }=5\times {10}^{-3}$ and grid mesh of ${dx}={dy}=0.151{\rm{\mu }}{\rm{m}}$. The dynamical parameters are such that the relaxation rates for the polariton state and exciton reservoir are ${\gamma }_{c}$ = 0.03 ps^−1^ and ${\gamma }_{r}$ = 1.5*γ*_*c*_, the polariton-polariton and polariton-exciton interaction strengths are ${g}_{c}$ = 5 × 10^−3^ meVµm^2^ and ${g}_{r}$ = 10 × 10^−3^ meVµm^2^, the amplification rate of the polariton state due to stimulated scattering of polariton from the reservoir is *R* = 3 × 10^−4^ ps^−1^µm^2^. Note that the radiative lifetime used in our simulations (${\gamma }_{c}$ = 0.03 ps^−1^, polariton linewidth ∆*E* ∼ 0.02 meV, polariton lifetime *τ*_*p*_ ∼ 30 ps) arises solely from photon escape and is typical for high-*Q* GaAs microcavities and common detunings. The same intrinsic decay rate was already employed in the topological lattice experiment of Klembt et al.^21^, on which also other parameters in this manuscript are based. While even longer photonic lifetimes of 100 − 270 ps in planar cavities with *Q* > 100,000^83–86^ are possible, achieving a linewidth of 0.02 meV in patterned microcavity lattices is also within experimental feasibility. Shallow etch-and-overgrowth techniques have enabled measured polariton linewidths of 0.3 meV and *Q*-factors of 7500 in patterned polariton structures with only 32 top distributed Bragg reflectors (DBRs)^87^, where these values were obtained under non-resonant excitation and without optimization for maximum *Q*.

In Fig. 3, the pump is given by a top-hat-like function of the time-interval *t*_0_ = 10[ℏ] and *t*_1_ = 850[ℏ] [see Fig. 3(b)]:

7 $${S}_{{\rm{pump}}}=\frac{1}{1+{e}^{-\frac{{t-t}_{0}}{2{\tau }_{t}}}}\left(1-\frac{1}{1+{e}^{-\frac{{t-t}_{1}}{2{\tau }_{t}}}}\right)\left(\begin{array}{c}{S}_{0,{\rm{pump}},+}{\bf{1}}\\ {S}_{0,{\rm{pump}},-}{\bf{1}}\end{array}\right)$$

with *τ*_*t*_ = 0.8[ℏ] and pump amplitude ${S}_{0,{\rm{pump}},\pm }={S}_{0,{\rm{pump}}}=2.5$ ps^−1^µm^−2^. The resonant probe is a continuous wave source, centered at $({x}_{s},{y}_{s})$ [see magenta star in Fig. 3a]

8 $${S}_{{\rm{probe}}}={e}^{-\frac{{\left({x}_{s}-x\right)}^{2}+{\left({y}_{s}-y\right)}^{2}}{2{\tau }_{{xy}}^{2}}}{e}^{-i{\omega }_{s}t}{e}^{i{k}_{s}x}\left(\begin{array}{c}{S}_{0,{\rm{probe}},+}{\bf{1}}\\ {S}_{0,{\rm{probe}},-}{\bf{1}}\end{array}\right)$$

with *τ*_*xy*_ = 1 µm, probe amplitude ${S}_{0,{\rm{probe}},\pm }={S}_{0,{\rm{probe}}}$ = 0.5 ps^−1^µm^−2^, resonant frequency ℏ*ω*_s_ = 0.35 meV, and resonant wavevector *k*_s_ = 0.6[*π/a*].

In Fig. 4, the probe takes the form

9 $${S}_{{\rm{probe}}}={e}^{-\frac{{\left({x}_{s}-x\right)}^{2}+{\left({y}_{s}-y\right)}^{2}}{2{\tau }_{{xy}}^{2}}}\left(\begin{array}{c}{S}_{0,{\rm{probe}},+}{\bf{1}}\\ {S}_{0,{\rm{probe}},-}{\bf{1}}\end{array}\right)\left({e}^{-i{\omega }_{s,1}t}{e}^{i{k}_{s,1}x}+{e}^{-i{\omega }_{s,2}t}{e}^{i{k}_{s,2}x}\right)$$

with $\tau_{xy}$ = 1 µm, probe amplitude ${S}_{0,{\rm{probe}},\pm }={S}_{0,{\rm{probe}}}=0.5$ ps^−1^µm^−2^, resonant frequencies $\hslash {\omega }_{s,1}=0.35\,{\rm{meV}}$ and $\hslash {\omega }_{s,2}=0.65\,{\rm{meV}}$, and resonant wavevectors *k*_s,1_ = 0.62[*π/a*] and *k*_s,2_ = 0.47[*π/a*].

## Supplementary information

Supplementary information for “Dynamically reconfigurable topological routing in nonlinear photonic systems”

## Data Availability

The data that support the findings of this study are available from the corresponding author upon reasonable request.
