# Supplementary material for: Dynamically reconfigurable topological routing in nonlinear photonic systems
Source: Light Sci Appl. 2026 Jan 3;15:46. doi: 10.1038/s41377-025-02108-1 (PMC12764893; doi:10.1038/s41377-025-02108-1)
Supplement: Supplementary file 1 — Supplementary information for “Dynamically reconfigurable topological routing in nonlinear photonic systems” [file 41377_2025_2108_MOESM1_ESM.pdf]

Supplementary information for "Dynamically reconfigurable topological routing in nonlinear photonic systems"

Stephan Wong,[1,*] Simon Betzold,[2] Sven Höfling,[2] and Alexander Cerjan[1]

[1] *Center for Integrated Nanotechnologies, Sandia National Laboratories, Albuquerque, New Mexico 87185, USA*
[2] *Julius-Maximilians-Universität Würzburg, Physikalisches Institut, and Würzburg-Dresden Cluster of Excellence ct.qmat, Lehrstuhl für Technische Physik, Am Hubland, Würzburg 97074, Germany*

(Dated: August 5, 2025)

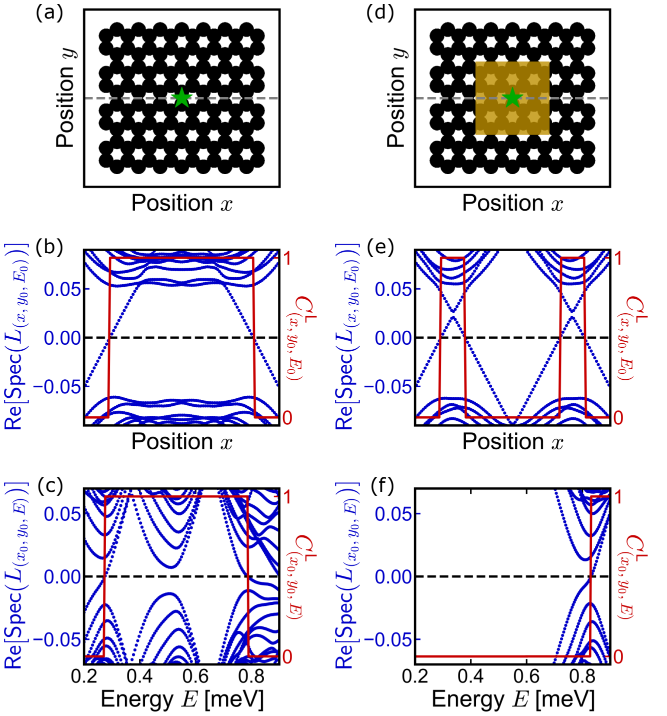

FIG. S1. **Probing topology of non-resonantly pumped systems using the spectral localizer framework.** (a) Potential landscape $V(\boldsymbol{x})$ of the polariton system arranged in a honeycomb lattice. The black and white regions correspond to potentials of $V = 6$ meV and $V = 0$ meV, respectively. (b) Eigenvalues of the spectral localizer $\mathrm{Re}\big[\mathrm{Spec}\big(L_{(x,y_0,E_0)}\big)\big]$ and the local Chern number $C^{\mathrm{L}}_{(x,y_0,E_0)}$ along the dashed line in (a) and at $E_0 = 0.35$ meV. (c) Eigenvalues of the spectral localizer $\mathrm{Re}\big[\mathrm{Spec}\big(L_{(x_0,y_0,E)}\big)\big]$ and the local Chern number $C^{\mathrm{L}}_{(x_0,y_0,E)}$ along the energy axis and at the position of green star in (a). (d)-(f) Same as (a)-(c) but with an yellow overlay on the lattice in (d), depicting the blueshift. Parameter values: lattice constant is $a = 2.95$ µm (center-to-center distance is 1.7 µm), radius of the rods is 1 µm; $m = 1.3\times10^{-4} m_0$ with m_0 the free electron mass, $\beta_{\mathrm{eff}} = 0.2$ meV µm^2, $\Delta_{\mathrm{eff}} = -0.3$ meV; blueshift energy used is $E_{\mathrm{blueshift}} = 0.55$ meV; $\kappa = 0.015$ meV µm^{-1}.

Supplementary note 1. PROBING THE TOPOLOGY IN POLARITON LATTICES WITH BLUESHIFT

In this section, we present how the system's local topology has been determined for a system with no blueshift and one that is partly blueshifted [see for example Figs. 2(b),(e),(h) and Figs. 2(d)-(f) in the main text]. The topology in the polariton lattice is characterized directly from the continuous model with the spectral localizer framework. In particular, the topology is probed by looking at the real parts of the spectral localizer's eigenvalues denoted $\mathrm{Re}\big[\mathrm{Spec}\big(L_{(x,y,E)}\big)\big]$, and the corresponding local Chern number $C^{\mathrm{L}}_{(x,y,E)}$.

In the absence of any nonlinearly induced blueshift [Fig. S1(a)], the lattice is topologically non-trivial. A real-space picture of the topology is shown in Figure S1(b) by plotting the spectral flow of the localizer $\mathrm{Re}\big[\mathrm{Spec}\big(L_{(x,y_0,E_0)}\big)\big]$ local Chern number $C^{\mathrm{L}}_{(x,y_0,E_0)}$ when varying x with fixed y_0 along the gray dashed line in Fig. S1, and at $E_0 = 0.35$ meV. The eigenvalues of the localizer cross the zero axis around the edges of the lattice, indicating a change of local Chern number and the non-trivial topology of the lattice. Similarly, the topology can also be spectrally resolved by varying (x_0, y_0, E) along the energy axis with a fixed position (x_0, y_0). Figure S1(c) displays $\mathrm{Re}\big[\mathrm{Spec}\big(L_{(x_0,y_0,E)}\big)\big]$ and $C^{\mathrm{L}}_{(x_0,y_0,E)}$, for a fixed spatial position (x_0, y_0) given by the green start in Fig. S1(a).

With a finite partial blueshift of the lattice [Fig. S1(d)], $B = E_{\mathrm{blueshift}} I$ and $\Gamma = 0$, the topology is only changed locally. By looking at the spectral localizer's spectrum along the dashed line, we can see a change of the topology inside the blueshifted region: There is additional crossing of zero for $\mathrm{Re}\big[\mathrm{Spec}\big(L_{(x,y_0,E_0)}\big)\big]$ and the local Chern number becomes trivial inside of the pumped region, as shown in Fig. S1(e). Moreover, the topology along the energy axis is also modified inside the blueshifted region. The local Chern number is now trivial up to approximately $E = 0.8$ meV as shown in Fig. S1(f), while there was non-trivial local Chern number for $E = 0.3-0.8$ meV in the unpumped system [see Fig. S1(c)].

The local Chern numbers shown in Fig. 1 in the main text correspond to the calculated local Chern number along the energy axis in Figs. S1(c),(f).

* Email: stewong@sandia.gov

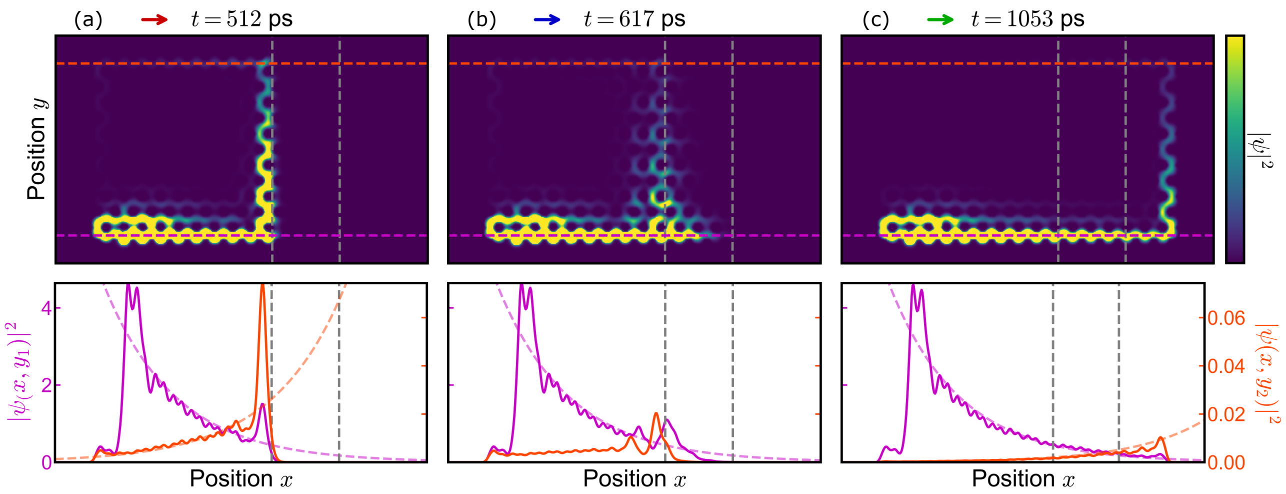

FIG. S2. **Propagation decay.** (a)-(c) Snapshot of the total intensity of the polariton $|\psi|^2 = |\psi_+|^2 + |\psi_-|^2$, with one-dimensional (1D) slice plots that show the intensities as the excited polariton propagates along the bottom (magenta) and top (orange) boundaries, respectively at y_1 (magenta dashed line) and y_2 (orange dashed line). The intensity plots use the same color scale, and the gray dashed lines indicate the non-resonant pump pattern. In the 1D slice plots, the dashed lines correspond to exponential curves e^{-x/ξ_p} with decay length ξ_p. The parameter values for the Hamiltonian, and the dynamics are the same as in Fig. 3 in the main text.

Supplementary note 2. PROPAGATION DECAY DUE TO INTRINSIC POLARITON LOSS

This section contains additional information about the propagation decay of an exciton-polariton.

Figure S2 plots the intensities as the excited polariton propagates along the bottom (magenta) and top (red) boundaries. The intensities decay exponentially due to the polariton lifetime, $\tau_p \approx 30$ ps, and can thus be fitted by an exponential e^{-x/ξ_p} of decay length given by $\xi_p = v_g \tau_p \approx 11.7$ µm^{-1}, where the group velocity $v_g \approx 0.35$ µm ps^{-1} can be found in the ribbon band structure in Fig. 2 in the main text.

Supplementary note 3. RECONFIGURABLE TOPOLOGY ROBUST TO DISORDER

This section demonstrates that the newly formed topological interfaces are robust against disorder. In particular, disorder in the position of the quantum wells and in the polariton potential are considered.

For example, for a 10% disorder in position (drawn from a uniform distribution), the dynamics of the topological edge states and the system's local Chern topology is plotted in Fig. S3(a)-(b), thus showing the robustness of the newly-formed topological interface (and of the topological edge states) against shift in position of the quantum wells. Similarly, one can show in Fig. S3(c)-(d) that newly-formed topological interface and of the topological edge states are also robust against perturbation in the polariton potential, with perturbation strength of 0.3 meV drawn from a uniform distribution.

Supplementary note 4. RECONFIGURABLE TOPOLOGICAL ROUTING WITH SMALL BLUESHIFT

Here, we show that the proposed method for reconfigurable topological routing also works if the blueshifted region becomes gapless in the energy range of interest. In particular, we discuss the case of a small blueshift, leading to an interface between a topological non-trivial gapped region with a topologically trivial system that is gapless in the same energy range.

In the absence of blueshift, the lattice is topologically non-trivial and its band structure and topology are similar to Figs. 2(a)-(c) in the main text [see Figs. S4(a)-(c)]. However, with a small blueshift of $E_{\text{blueshift}} = 0.1$ meV, $\Gamma = 0$, depicted by the yellow overlay in Fig. S4(d), the bands are only slightly blueshifted, resulting in the bulk bands spectrally overlapping with the energy of interest [see gray shaded area in Fig. S4(e)]. Thus, this partially blueshifted system is gapless. Nevertheless, even though the system is gapless in the relevant energy range, the topology can still be classified using the spectral localizer [1, 2]. The right panel of Fig. S4(e) shows the local Chern number, calculated using similar methods as in Sect. Supplementary note 1, demonstrating that the blueshifted portion of the system is topologically trivial in part of the gray shaded energy range. Therefore, a bulk-edge correspondence is possible at the interface between the blueshifted and non-blueshifted regions. Although the system is gapless, namely there is no complete shared band gap in the nonlinear heterostructure, the system features an incomplete band gap where the topological mode appears [Fig. S4(h)] due to the change

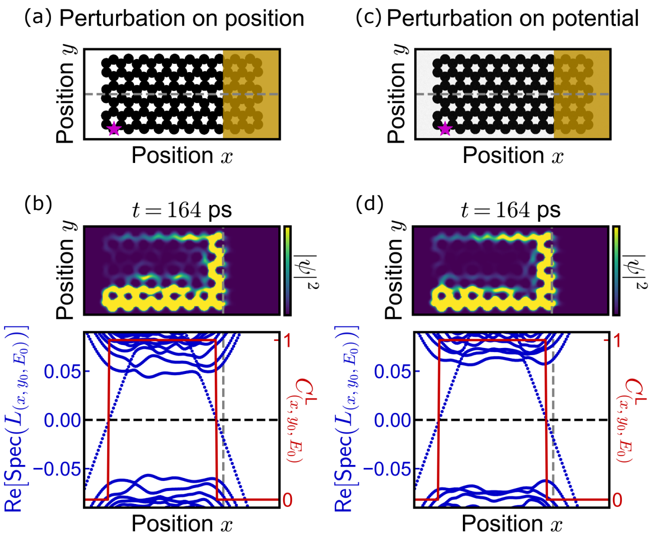

FIG. S3. **Topological robustness for nonlinear-induced topological interfaces.** (a) Potential landscape $V(\boldsymbol{x})$ of the polariton system arranged in a honeycomb lattice, with 10% perturbation on the quantum wells' position. The black and white regions correspond to potentials of $V = 6$ meV and $V = 0$ meV, respectively. The yellow shaded area depicts the pump pattern, the magenta star indicates the position of the probe source. (b) Snapshot of the total intensity of the polariton $|\psi|^2 = |\psi_+|^2 + |\psi_-|^2$, with the corresponding eigenvalues of the spectral localizer $\mathrm{Re}\big[\mathrm{Spec}\big(L_{(x,y_0,E_0)}\big)\big]$ and the local Chern number $C^{\mathrm{L}}_{(x,y_0,E_0)}$ along the gray dashed line in (a) and at $E_0 = 0.35$ meV. (c)-(d) Same as (a)-(b) but with perturbation on the polariton potential of strength 0.3 meV. The parameter values for the Hamiltonian, and the dynamics are the same as in Fig. 3 in the main text.

of the local topology across the interface. These topological edge modes are dubbed "topological edge mode resonances". The red (and green) lines correspond to the topological mode localized at the newly formed topological interface (and bottom edge of the lattice), as shown through the LDOS of the red line at $E = 0.33$ meV plotted in Fig. S4(i).

The dynamic behavior of the nonlinearly induced gapless topological heterostructure is shown in Fig. S5. The calculation of the local Chern number along the gray dashed line [Fig. S5(a)] illustrates the change of topology at $E_0 = 0.33$ meV inside gapless energy range. The system becomes trivial inside the pumped region, as calculated at the final time (t_f). Consequently, using a resonant probe source with a frequency and wavevector chosen in the incomplete band gap (see caption of Fig. S5), a topological resonance can be excited that propagates along the newly topological interface, as shown in Fig. S5(b). Here, the gapless nature of the trivial region means that the propagating edge mode is a resonance, and has some overlap with the available bulk states yielding another source of loss for this channel; indeed, some diffraction into the bulk can be seen in

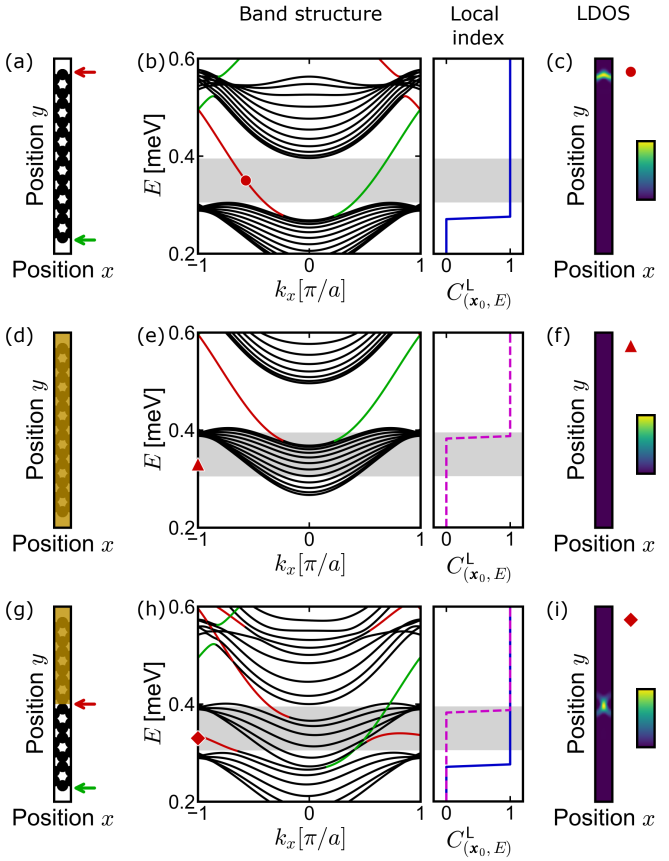

FIG. S4. **Change of the topology due to the blueshift, gapless case.** (a) Potential landscape $V(\boldsymbol{x})$ of the ribbon polariton lattice arranged in a honeycomb lattice. The black and white region correspond to a potential of $V = 6$ meV and $V = 0$ meV, respectively. (b) Ribbon band structure of (a), and the corresponding local Chern number $C^{\mathrm{L}}_{(\boldsymbol{x}_0,E)}$, at a the given energy, calculated using the spectral localizer. In the band structure, the black lines correspond to the bulk modes, and the green (red) lines denotes the topological edge mode dispersion localized at the bottom (top) side of the lattice, as shown by the color-coded arrows in (a). The gray shaded area indicates the first band gap, which is the energy range of interest. (c) Local density of states (LDOS) of the red line at $E = 0.35$ meV, $k_x = -0.6[\pi/a]$. (d)-(f) Same as (a)-(c) but with an yellow overlay depicting the blueshift of the whole ribbon structure. In (f), the LDOS is plotted for $E = 0.33$ meV, $k_x = -1[\pi/a]$. (g)-(i) Same as (a)-(c) but with the blueshift applied only to half of the ribbon lattice. The red (and green) lines in the band structure correspond to the topological edge modes localized at the interface between the blueshifted and non-bleushifted areas (and at the bottom edge of the lattice). In (h), the solid blue line and dashed magenta line are the local Chern number calculated inside the non-blueshifted and blueshifted regions, respectively. (i) LDOS of the red line at $E = 0.33$ meV, $k_x = -1[\pi/a]$. Parameter values: The lattice constant is $a = 2.95$ µm (center-to-center is 1.7 µm), radius of the rods is 1 µm; $m = 1.3 \times 10^{-4} m_0$ with m_0 the free electron mass, $\beta_{\mathrm{eff}} = 0.2$ meV µm^2, $\Delta_{\mathrm{eff}} = -0.3$ meV; blueshift energy used is $E_{\mathrm{blueshift}} = 0.1$ meV; $\kappa = 0.02$ meV µm^{-1}.

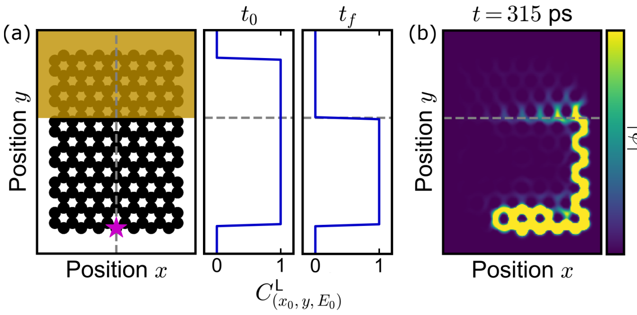

FIG. S5. **Reconfigurable topological routing with non-resonant pumping, gapless case.** (a) Potential landscape $V(\boldsymbol{x})$ of the polariton lattice arrange in a honeycomb lattice, and the local Chern number $C^{\rm L}_{(\boldsymbol{x},E)}$ along the x_0 gray dashed line and at $E = 0.33$ meV, calculated using the spectral localizer before the pump starts (t_i) and at the final time (t_f). The yellow shaded area depicts the pump pattern, the magenta star indicates the position of the probe source. (b) Snapshot of the total intensity of the polariton $|\psi|^2 = |\psi_+|^2 + |\psi_-|^2$. The parameter values for the Hamiltonian are the same as in Fig. S4. Dynamical parameter values: $\gamma_c = 0.03$ ps^{-1}, $\gamma_r = 1.5\gamma_c$, $g_c = 5 \times 10^{-3}$ meV µm^2, $g_r = 10 \times 10^{-3}$ meV µm^2, $R = 3 \times 10^{-4}$ ps^{-1}µm^2; $S_{0,\rm pump} = 0.5$ ps^{-1}µm^{-2}, $S_{0,\rm probe} = 0.5$ ps^{-1}µm^{-2}; $\hbar\omega_{\rm s} = 0.35$ meV, $k_{\rm s} = 1[\pi/a]$; $\kappa = 0.015$ meV µm^{-1}.

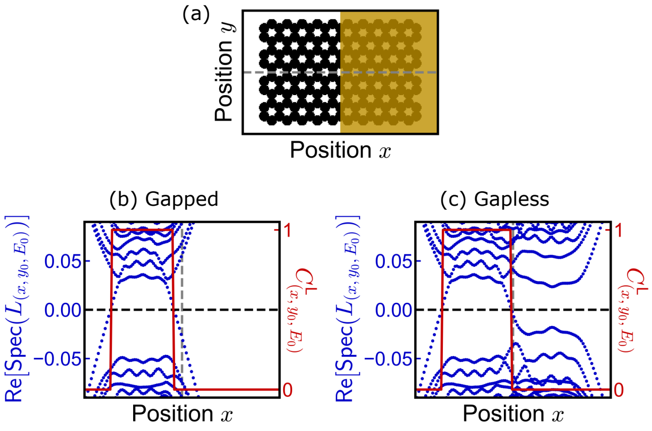

FIG. S6. **Spectrum of the spectral localizer for a gapped or gapless "heterostructure" interface.** (a) Potential landscape $V(\boldsymbol{x})$ of the polariton lattice. (b),(c) Eigenvalues of the spectral localizer $\mathrm{Re}\left[\mathrm{Spec}\left(L_{(x,y_0,E_0)}\right)\right]$ and the local Chern number $C^{\rm L}_{(x,y_0,E_0)}$ along the dashed line in (a) and at $E_0 = 0.325$ meV. The blueshift energy used for the gapped and gapless are $E_{\rm blueshift} = 0.55$ meV and $E_{\rm blueshift} = 0.1$ meV, respectively. The parameter values for the Hamiltonian are the same as in Fig. S1.

Fig. S5(b).

Note that the topological robustness of the topological edge mode resonances are expected to be similar to the topological edge modes obtained from a interface between gapped regions, as shown in Fig. S6. Indeed, the topological robustness characterized by local gap, which is the smallest eigenvalue (in absolute value) of the spectral localizer is similar for both the gapped and gapless case within the $C^{\rm L} = 1$ region, which is the (topological non-trivial) region that guarantees the existence of the topological edge mode. In this case, a smaller local gap in the $C^{\rm L} = 0$ region does not affect the topological edge mode, as perturbations within this region is not expected to change the trivial topology, meaning the topological interface remains. Notably, while the topological edge modes and topological edge mode resonances have similar topological robustness, the greater overlap of the topological mode resonances with the bulk modes leads to an increased effective intrinsic loss, which is further increased in presence of disorder due to additional leakage to the bulk channels.

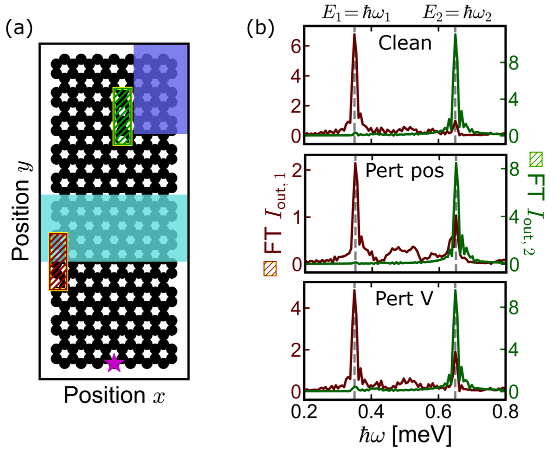

FIG. S7. **Frequency-dependent reconfigurable topological routing with non-resonant pumpings in presence of disorder.** (a) Potential landscape $V(x, y)$ of the polariton lattice arrange in a honeycomb lattice. The cyan (blue) shaded area depicts the pump pattern with pump amplitude $S^{(0)}_{0,\rm pump}$ ($S^{(1)}_{0,\rm pump}$), the magenta star indicates the position of the probe source. (b) Fourier transform (FT) of the polariton signal over the red and green hatched areas in (a) for the clean system and with perturbation in the quantum wells' position of 10% and in the polariton potential of strength of 0.3 meV. The parameter values for the Hamiltonian and for the dynamics are the same as in Fig. 4.

Supplementary note 5. ROBUSTNESS OF MULTI-CHANNEL TOPOLOGICAL ROUTING IN PRESENCE OF DISORDER

This section demonstrates the robustness of the multi-channel configuration against perturbation. Here, we consider perturbation in the quantum wells' position up to a 10% shift, and perturbation in the polariton potential up to 0.3 meV.

Figure S7(b) shows, for the clean and perturbed system, the Fourier transform (FT) of the polariton time-evolution summed over the different output-channel areas [see the red and green hatched area in Fig. S7(a)]. The

respective peaks at energies E_1 and E_2 in the clean system reveal the multi-routing feature of the system where excited polariton mode at energy E_1 is routed toward the red hatched area, while the the excited polariton at E_2 is routed toward the green hatched area. In presence of perturbation, the red curve originating from the red hatched area pick an additional lower peak at E_2. This peak at E_2 is coming from a E_2-mode being partly re-routing at the cyan interface, but this mode is not expected to be robust as the cyan interface is not topological at energy E_2. Nevertheless, when perturbation is introduced, the respective peaks at E_1 and E_2 remains, thus illustrating the robustness of the multi-channel topological routing in presence of disorder.

Supplementary note 6. EXPERIMENTAL REALIZATION SCHEME

The envisioned experiment would employ a two-dimensional lattice of coupled micropillar cavities, fabricated from a GaAs-based microcavity structure with embedded quantum wells, similar to those used in previous demonstrations of polaritonic topological insulators [3]. The lattice geometry, typically honeycomb, would be chosen to support topological phases under appropriate symmetry-breaking perturbations, such as TE-TM splitting, magnetic fields, or staggered sublattice potentials.

Micropillars would have diameters in the range of $2.0 - 3.0$ µm, with center-to-center distances of approximately $1.6 - 2.7$ µm, depending on the pillar size, to ensure sufficient coupling between adjacent sites. The system would be excited non-resonantly using a continuous-wave laser beam tuned above the exciton resonance (e.g., 740 nm), thereby creating an incoherent exciton reservoir. The spatial profile of the pump would be structured using a spatial light modulator (SLM), enabling site-selective and dynamically reconfigurable injection of the exciton population. This reservoir modifies the polariton effective potential locally via repulsive interactions, allowing control over onsite energies and, consequently, the topological phase of different regions within the lattice.

The pump profile would be engineered to induce sharp boundaries between topologically trivial and nontrivial regions within a single, continuous lattice. The SLM's spatial modulation resolution would be matched to the lattice scale using appropriate optics. Real-time dynamic reconfiguration of the domain wall could be achieved by updating the SLM pattern.

The edge state propagation would be probed using near-field photoluminescence imaging, collected through a microscope objective and projected onto a CCD camera. Momentum-space and energy-resolved measurements could be performed via Fourier-space imaging and spectral filtering, respectively. The time-resolved measurements using a streak camera would allow observation of edge state dynamics during topological transitions induced by time-varying pump profiles.

The topological characterization would rely on numerical evaluation of the spectral localizer from the measured or simulated system Hamiltonian, enabling a local, real-space determination of topological invariants, even in the presence of dissipation and inhomogeneous pumping. This framework is particularly well-suited to non-Hermitian, driven-dissipative systems and can be directly applied to validate the formation and manipulation of topological boundaries in the experiment.

This experimental design enables the observation of dynamically reconfigurable topological states in a photonic platform without requiring material modification or slow electro-optic control, relying instead on fast, reprogrammable optical pumping patterns to manipulate topological phases in real time.

Supplementary note 7. TOPOLOGICAL ROUTING WITH CIRCULAR POLARIZED PUMP PATTERN

So far, we have considered polariton lattices that are topologically non-trivial at the energy range of interest in the absence of a nonlinearly induced blueshift. However, with a gapless topologically trivial polariton lattice, namely without any external magnetic field $\Delta_{\text{eff}} = 0$ meV, the existence of topological modes and the dynamical control of their propagation path can also be realized using non-resonant circularly polarized pumps.

A. Non-trivial topology induced by spin-dependent blueshift

Non-trivial topological polariton lattices can be realized solely using a circularly polarized pump [4–7]. Using a circularly polarized non-resonant pump will lead to nonlinear interactions only to one of the spin sectors, inducing a blueshift in the corresponding polariton spin sector [8, 9]. As a result, by redefining the reference polariton energy, an effective Zeeman splitting term $\tilde{\Delta}_{\text{eff}} = E_{\text{blueshift}}$ is achieved, known as optical Zeeman splitting, and can be used for inducing non-trivial topology

$$B = \begin{pmatrix} E_{\text{blueshift}} & 0 \\ 0 & 0 \end{pmatrix} = \begin{pmatrix} \tilde{E}_0 + \frac{1}{2}\tilde{\Delta}_{\text{eff}} & 0 \\ & \tilde{E}_0 - \frac{1}{2}\tilde{\Delta}_{\text{eff}} \end{pmatrix}, \tag{S1}$$

with $\tilde{E}_0 = E_{\text{blueshift}}/2$. Figure S8(a) illustrates this optical Zeeman splitting process. The landscape of the polariton potential is shown for both spin sectors, where a blueshift is only applied on the ψ_+-sector (shown in red shaded area). Similar to the previous band calculations, the blueshift is manually added to the ψ_+-subspace to emulate the positive circular pump, with $\Gamma = 0$. The corresponding ribbon band structure (left panel) and the local Chern number at the associated energy in (right

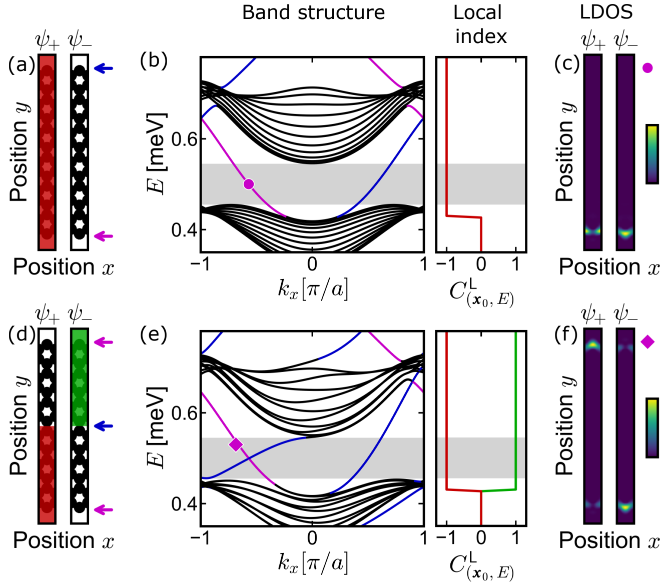

FIG. S8. **Change of the topology due to the spin-dependent blueshift.** (a) Potential landscape $V(\boldsymbol{x})$ of the ribbon polariton lattice for the two spin sectors ψ_+, ψ_-, arranged in a honeycomb lattice. The black and white regions correspond to a potential of $V = 6$ meV and $V = 0$ meV, respectively. The red overlay denotes the blueshift applied on a positive spin sector (ψ_+). (b) Ribbon band structure of (a), and the corresponding local Chern number $C^{\mathrm{L}}_{(\boldsymbol{x}_0,E)}$, at a the given energy, calculated using the spectral localizer. In the band structure, the black lines correspond to the bulk modes, and the magenta (blue) lines denotes the topological edge mode dispersion localized at the bottom (top) side of the lattice, as shown by the color-coded arrows in (a). The gray shaded area indicate the first band gap, which is the energy range of interest. (c) Local density of states (LDOS) of the magenta line at $E = 0.5$ meV, $k_x = -0.6[\pi/a]$. (d)-(f) Same as (a)-(c) but with the blueshift applied on both spin sectors and on opposite half of the ribbon lattice. The blue (and magenta) lines in the band structure correspond to the topological edge modes localized at the interface between the blueshifted areas (and at the edges of the lattice). In (e), the solid red and green lines are the local Chern number calculated inside the ψ_+-blueshifted and ψ_--blueshifted regions, respectively. (f) LDOS of the magenta line at $E = 0.53$ meV, $k_x = -0.72[\pi/a]$. Parameter values: lattice constant is $a = 2.95$ µm (center-to-center is 1.7 µm), radius of the rods is 1 µm; $m = 1.3 \times 10^{-4} m_0$ with m_0 the free electron mass, $\beta_{\mathrm{eff}} = 0.2$ meV µm^2, $\Delta_{\mathrm{eff}} = 0$ meV; spin-dependent blueshift energy used is $E_{\mathrm{blueshift}} = 0.3$ meV; $\kappa = 0.01$ meV µm^{-1}.

panel) is plotted in Fig. S8(b), demonstrating the opening of topological band gap with a local Chern number $C^{\mathrm{L}}_{(\boldsymbol{x}_0,E)} = -1$. The magenta (blue) lines in the band structure depict the topological edge modes localized at the top (bottom) lattice edge, as shown by the LDOS of the magenta line at $E = 0.5$ meV in Fig. S8(c). Note that similar results can be realized by applying a blueshift on the ψ_--subspace, with the difference being that the local Chern number will be opposite, $C^{\mathrm{L}}_{(\boldsymbol{x}_0,E)} = 1$.

With a ψ_+-blueshift on one part of the lattice and a ψ_--blueshift on the other part of the lattice, an internal topological interface can be created. In particular, these blueshifts can be realized by illuminating the sample with a left (or right) circular polarized non-resonant pump in one part of the lattice (or the other), as shown in Fig. S8(d) where the red (green) shaded area denotes the induced blueshift on the ψ_+ (ψ_-) subspace. Such opposite circular polarized pump configuration will lead to topological modes at the interface between the $C^{\mathrm{L}}_{(\boldsymbol{x}_0,E)} = -1$ and $C^{\mathrm{L}}_{(\boldsymbol{x}_0,E)} = 1$ regions of the lattice. Consequently, a new topological interface is formed with a local Chern number difference of $|\Delta C^{\mathrm{L}}_{(\boldsymbol{x}_0,E)}| = 2$, resulting in two topological edge modes at the interface [see Fig. S8(e)]. Note that the magenta line in the ribbon band structure [see left panel of Fig. S8(e)] is doubly degenerate, and its associated LDOS is plotted in Fig. S8(f). Specifically, the ψ_+ (ψ_-) spin sector has higher LDOS amplitude on the top (bottom) edge of the lattice.

B. Probing the topology in polariton lattices with spin-dependent blueshift

Here, we present how topology has been studied for a polaritonic system with no external magnetic, and with a blueshift on a single spin sector [see for example Figs. S8(b),(e) in the main text], with $\Gamma = 0$. The topology in the polariton lattice is characterized directly from the continuous model with the spectral localizer, similar to what is discussed in Sect. Supplementary note 1.

In the absence of non-resonant pump, namely without any added blueshift, the system is topologically trivial, as shown in Fig. S9(b),(c). In particular, even though the system is gapless because of the lack of external magnetic field, and lacks time-reversal symmetry breaking, its topology can still be calculated with the spectral localizer and the local Chern number. Figure S9(b) shows that the local Chern number is zero along the dashed line in Fig. S9(a) at $E_0 = 0.5$ meV, and Figure S9(c) indicates that the system is trivial at least up to 1 meV.

With opposite non-resonant circular polarized pumps, as depicted with green and red overlays in Fig. S9(d), opposite optical Zeeman effects arise in different regions of the lattice. Figure S9(e) plots the spectral flow of $L_{(x_0,y,E_0)}$ and the local Chern number $C^{\mathrm{L}}_{(x_0,y,E_0)}$ along the dashed in Fig. S9(d) at $E_0 = 0.5$ meV, revealing the system is topologically non-trivial and that the upper and lower halves of the lattice possess opposite local Chern numbers. Looking at the topology at the location of the blue star (blue diamond) [see Fig. S9(d)], the top (bottom) panel of Fig. S9(f) indicates the system is non-trivial for some energy range starting from around 0.4 meV with local Chern number $C^{\mathrm{L}}_{(x_0,y_0,E)} = 1$ ($C^{\mathrm{L}}_{(x_0,y_1,E)} = -1$).

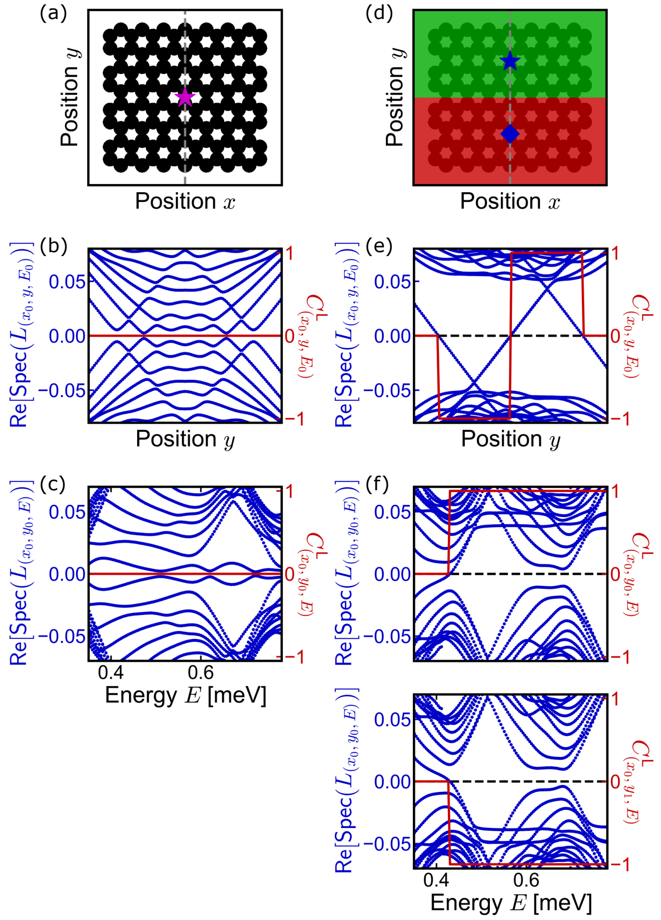

FIG. S9. **Probe of the topology with the spectral localizer for systems with non-resonant circular polarized pump.** (a) Potential landscape $V(\boldsymbol{x})$ of the polariton lattice arranged in a honeycomb lattice. The black and white regions correspond to a potential of $V = 6$ meV and $V = 0$ meV, respectively. (b) Eigenvalues of the spectral localizer $\mathrm{Re}\big[\mathrm{Spec}\big(L_{(x_0,y,E_0)}\big)\big]$ and the local Chern number $C^{\mathrm{L}}_{(x_0,y,E_0)}$ along the dashed line in (a) and at $E = 0.5$ meV. (c) Eigenvalues of the spectral localizer $\mathrm{Re}\big[\mathrm{Spec}\big(L_{(x_0,y_0,E)}\big)\big]$ and the local Chern number $C^{\mathrm{L}}_{(x_0,y_0,E)}$ along the energy axis and at the position of the magenta star in (a). (d)-(f) Same as (a)-(c) but with a red and green overlay on the lattice in (d), depicting the blueshift on the ψ_+ and ψ_- spin sectors. The top and bottom panels of (f) are calculated at the loaction of the blue star (x_0, y_0) and blue diamond (x_0, y_1), respectively. Parameter values: lattice constant is $a = 2.95$ µm (center-to-center is 1.7 µm), radius of the rods is 1 µm; $m = 1.3 \times 10^{-4} m_0$ with m_0 the free electron mass, $\beta_{\mathrm{eff}} = 0.2$ meV µm^2, $\Delta_{\mathrm{eff}} = 0$ meV; spin-dependent blueshift energy used is $E_{\mathrm{blueshift}} = 0.3$ meV; $\kappa = 0.01$ meV µm^{-1}.

The local Chern numbers shown in Fig. S8 correspond to the calculated local Chern number along the energy axis in Figs. S1(c),(f).

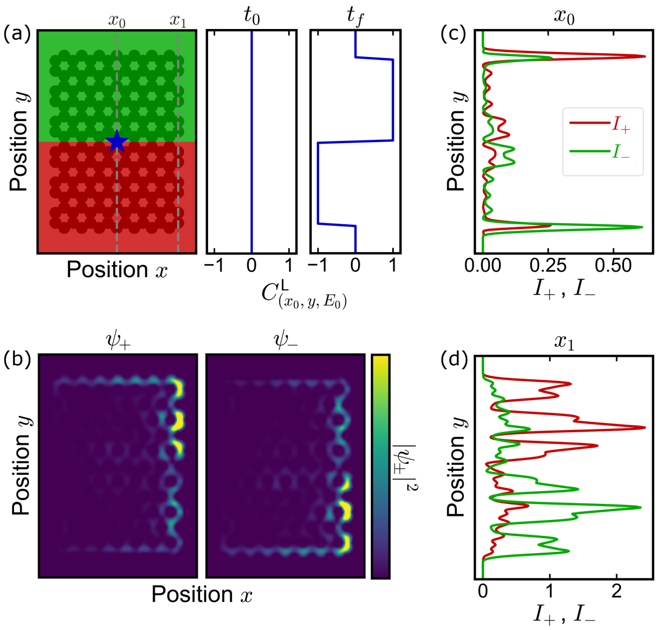

FIG. S10. **Reconfigurable topological routing with non-resonant circular polarized pumping.** (a) Potential landscape $V(\boldsymbol{x})$ of the polariton lattice arranged in a honeycomb lattice, and the local Chern number $C^{\mathrm{L}}_{(x_0,y,E_0)}$ along the x_0 gray dashed line and at $E_0 = 0.5$ meV, calculated using the spectral localizer before the pump starts (t_i) and at the final time (t_f). The red and green overlays depict the positive $(S_{\mathrm{pump},+})$ and negative $(S_{\mathrm{pump},-})$ circular polarized pump, respectively. The blue star indicate the position of the probe source. (b) Snapshot of the intensity of the polariton for each spin sectors $|\psi_+|^2$, $|\psi_-|^2$ at $t = 455[\hbar] \approx 300$ ps. (c)-(d) Integrated intensity of each spin sector over a width of 1 µm in the y-direction, at the positions given by the red and green crosses in (a), along the x_0 and x_1 gray dashed line in (a). The parameter values for the Hamiltonian are the same as in Fig. S8. Dynamical parameter values: $\gamma_c = 0.05$ ps^{-1}, $\gamma_r = 1.5\gamma_c$, $g_c = 5 \times 10^{-3}$ meV µm^2, $g_r = 10 \times 10^{-3}$ meV µm^2, $R = 3 \times 10^{-4}$ ps^{-1}µm^2; $S_{0,\mathrm{pump}} = 2.3$ ps^{-1}µm^{-2}, $S_{0,\mathrm{probe}} = 0.5$ ps^{-1}µm^{-2}; $\hbar\omega_{\mathrm{s}} = 0.5$ meV, $k_{\mathrm{s}} = 0.5[\pi/a]$; $\kappa = 0.01$ meV µm^{-1}.

C. Topological routing

To illustrate topological routing using the optical Zeeman effect, we consider the dynamic response of the system shown in Fig. S10(a). The red and green overlays on the polariton potential denote the pump pattern with positive and negative circular polarized non-resonant pumps, inducing a ψ_+-blueshift and ψ_--blueshift respectively. In particular, the dynamics of system is calculated using the modified rate equations [Eqs. (1)-(2)] from the continuous model. Additionally, two slowly increasing and circularly polarized non-resonant pumping sources are used, as depicted in Fig. S10 with the red and green overlays, each with maximum amplitudes $S_{0,\mathrm{pump}} = 2.3$ ps^{-1}µm^{-2} below the condensate threshold; and a resonant Gaussian source with amplitude

$S_{0,\mathrm{probe}} = 0.5$ ps^{-1}µm^{-2}, located at the blue star [see Fig. S10(a)], is used to excite the topological modes. Note that, without loss of generality, the spin relaxation is here not included in our model [9, 10], although the main effect will be to increase the non-resonant pump powers to achieve the same desired optical Zeeman strength. See the Section Supplementary note 8 for additional information.

The resulting dynamics of the change in real-space topology from the difference in the local Chern number [see the right panels of Fig. S10(a)] demonstrates the emergence of a topological interface in the interior of the lattice and thus the existence of topological modes. At the initial time (t_0), before the lattice is illuminated, the topology of the system is trivial with local Chern number being $C^{\mathrm{L}}_{(x_0,y,E_0)} = 0$ all along the gray dashed and at $E_0 = 0.5$ meV. At the final simulated time (t_f), under circular polarized illumination, the system is topologically non-trivial with local Chern numbers being $C^{\mathrm{L}}_{(x_0,y,E_0)} = -1$ and $C^{\mathrm{L}}_{(x_0,y,E_0)} = 1$ in the lower and upper half of the lattice, respectively, in according with the polarized pump patterns. Figure S10(b) shows a time snapshot of the intensities $|\psi_+|^2$, $|\psi_-|^2$ for each spin sector, demonstrating the excitation and propagation of the topological mode along the newly formed topological interface. After reaching the right edge of the lattice, the topological mode is split into an upward and downward propagating topological mode along the outer topological interface from the lattice edge.

Remarkably, at the bifurcation of different topological interfaces, a finite spin separation occurs. In particular, when the excited topological modes reached the right edge of the lattice and split into upward and downward propagating topological modes [see Fig. S10(b)], ψ_+ polariton states are dominant in the upper half of the lattice, and vice versa for the ψ_- states. This spin separation is quantified by looking at the integrated intensities I_+, I_- over finite width of 3 µm centered around x_0 and x_1 [see dashed line sin Fig. S10(a)], as plotted in Figs. S10(c)-(d). At x_1 [see Fig. S10(d)], on the right lattice edge, the spin up intensity I_+ is predominant in the upper half lattice edge, while the spin down I_- is higher on the lower half. At x_0 [see Fig. S10(c)], the topological edge mode along the top (or bottom) has higher I_+ (or I_-). Note that similar topological mode propagation can be achieved when combined with a linearized polarized pump, inducing an internal trivial gapped region and topological interface, akin to the outer edge of the lattice. Consequently, using a linearly polarized pump in addition to particular circularly polarized pump patterns provides a possible control mechanism for topological modes, as well as over the position where the modes bifurcate and the spin separation arises.

In Figure S10, the pump is given by

$$S_{\mathrm{pump}} = \begin{cases} S_{0,\mathrm{pump}} \frac{1}{1+e^{-\frac{t-t_0}{2\tau}}} \begin{pmatrix} \mathbf{1} \\ \mathbf{0} \end{pmatrix} & \text{for } y \text{ in lower half} \\ S_{0,\mathrm{pump}} \frac{1}{1+e^{-\frac{t-t_0}{2\tau}}} \begin{pmatrix} \mathbf{0} \\ \mathbf{1} \end{pmatrix} & \text{for } y \text{ in upper half} \end{cases}, \tag{S2}$$

with $t_0 = 10[\hbar]$, $\tau_t = 0.9[\hbar]$, and the resonant probe is a Gaussian source, located at $(x_{\mathrm{s}}, y_{\mathrm{s}}) = (0, 0)$ [see blue star in Fig. S10(a)] and centered at a time $t_0 = 300[\hbar]$,

$$S_{\mathrm{probe}} = S_{0,\mathrm{probe}} e^{-\frac{(x_{\mathrm{s}}-x)^2+(y_{\mathrm{s}}-y)^2}{2\tau_{xy}^2}} e^{-\frac{(t-t_0)^2}{2\tau_t^2}} \times e^{-i\omega_{\mathrm{s}}t} e^{ik_{\mathrm{s}}x} \begin{pmatrix} \mathbf{0} \\ \mathbf{1} \end{pmatrix}, \tag{S3}$$

with $\tau_{xy} = 1$ µm, $\tau_t = 40[\hbar]$.

Supplementary note 8. EFFECT OF SPIN RELAXATION FOR THE OPTICAL ZEEMAN SPLITTING

As the optical Zeeman effect relies on the spins of the exciton reservoir $n_r(\boldsymbol{x}, t) = [n_{r,+}(\boldsymbol{x}, t), n_{r,-}(\boldsymbol{x}, t)]$, this section studies the effect of considering spin relaxations in the rate equations. The spin relaxation is included by considering the couplings J between the different spins in the exciton reservoir. The rate equations are therefore slightly modified as [9]

$$i\hbar\frac{\partial}{\partial t}\boldsymbol{\psi} = H_0\boldsymbol{\psi} - i\hbar\frac{\gamma_c}{2}\boldsymbol{\psi} + g_c|\boldsymbol{\psi}|^2\boldsymbol{\psi} + \left(g_r + i\hbar\frac{R}{2}\right)n_r\boldsymbol{\psi} + S_{\mathrm{probe}}, \tag{S4}$$

$$\frac{\partial}{\partial t}n_{r,\pm} = -\left(\gamma_r + R|\boldsymbol{\psi}_\pm|^2\right)n_{r,\pm} + J(n_{r,\mp} - n_{r,\pm}) + S_{\mathrm{pump},\pm}, \tag{S5}$$

where H_0 is the Hamiltonian and denotes the kinetic energy and the couplings between of the polaritons, γ_c and γ_r are the relaxation rates for the polariton state and exciton reservoir, g_c and g_r are the polariton-polariton and polariton-exciton interaction strengths, R is the amplification rate of the polariton state due to stimulated scattering of polariton from the reservoir, $S_{\mathrm{probe}}(\boldsymbol{x}, t)$ is the resonant probe for exciting the polaritons, and $S_{\mathrm{pump}}(\boldsymbol{x}, t)$ is the non-resonant pump for exciting the free carriers.

One of the main consequences of spin relaxation is the pump power used to achieved a given blueshift on the spin sectors. Indeed, from Eq. (S5), the steady states of

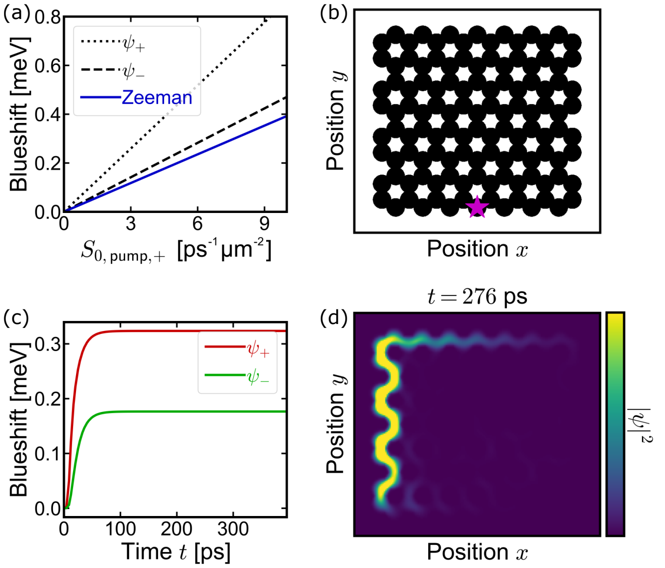

FIG. S11. **Optical Zeeman effect with spin relaxations.** (a) Theoretical blueshift from the steady state solution of the ψ_+ and ψ_- density exciton reservoir in the rate equations [Eq. (S5)], with $S_{0,\text{pump},-} = 0$ ps^{-1}µm^{-2}. Blue line is the effect of the Zeeman splitting, calculated from the difference between the ψ_+ and ψ_- blueshift. (b) Potential landscape $V(\boldsymbol{x})$ of the polariton lattice arranged in a honeycomb lattice. The magenta star indicate the position of the probe source. (c) Temporal evolution of the spin-dependent blueshift. (d) Snapshot of the total intensity of the polariton $|\psi|^2 = |\psi_+|^2 + |\psi_-|^2$. The parameter values for the Hamiltonian are the same as in Fig. S9. Dynamical parameter values: $\gamma_c = 0.05$ ps^{-1}, $\gamma_r = 1.5\gamma_c$, $g_c = 5 \times 10^{-3}$ meV µm^2, $g_r = 10 \times 10^{-3}$ meV µm^2, $R = 3 \times 10^{-4}$ ps^{-1}µm^2, $J = 0.09$ ps^{-1}; $S_{0,\text{pump},+} = 7.5$ ps^{-1}µm^{-2}, $S_{0,\text{pump},-} = 0$ ps^{-1}µm^{-2}, $S_{0,\text{probe},\pm} = 0.5$ ps^{-1}µm^{-2}; $\hbar\omega_s = 0.86$ meV, $k_s = 0.5[\pi/a]$.

the density of the exciton reservoir reads

$$n_{r,-,\text{th}} = \frac{J}{\gamma_r(\gamma_r + 2J)} S_{\text{pump},+} + \frac{\gamma_r + J}{\gamma_r(\gamma_r + 2J)} S_{\text{pump},-}, \tag{S6}$$

$$n_{r,+,\text{th}} = \frac{1}{\gamma_r + J} S_{\text{pump},+} + \frac{J}{\gamma_r + J} n_{r,-,\text{th}}. \tag{S7}$$

As a result, by redefining the reference polariton energy, an effective Zeeman splitting term $\tilde{\Delta}_{\text{eff}} = E_{\text{blueshift},+} - E_{\text{blueshift},-}$ is achieved and the the overall system is blueshifted by $\tilde{E}_0 = (E_{\text{blueshift},+} + E_{\text{blueshift},-})/2$

$$\begin{aligned} B &= \begin{pmatrix} E_{\text{blueshift},+} & 0 \\ 0 & E_{\text{blueshift},-} \end{pmatrix} \\ &= \begin{pmatrix} \tilde{E}_0 + \frac{1}{2}\tilde{\Delta}_{\text{eff}} & 0 \\ & \tilde{E}_0 - \frac{1}{2}\tilde{\Delta}_{\text{eff}} \end{pmatrix}. \end{aligned} \tag{S8}$$

Thus, injecting single spin excitons will lead to a finite density of the other exciton spin as well, meaning that higher pump powers are required to achieve the same amount of blueshift as in the case of neglected spin relaxation. According to Fig. S11(a), a blueshift of $\tilde{\Delta}_{\text{eff}} = 0.3$ meV needs a pump power of $S_{0,\text{pump},+} = 7.5$ ps^{-1}µm^{-2}, $S_{0,\text{pump},-} = 0$ ps^{-1}µm^{-2}, compared to $S_{0,\text{pump}} = 2.3$ ps^{-1}µm^{-2} in Sect. Supplementary note 7 C when neglecting the spin relaxation.

Figures S11(b)-(d) shows the dynamics of the system using the modified rate equations [Eqs.(S4)-(S5)]. In particular, a non-resonant positive circularly polarized pump is illuminating the whole lattice (the overlay of the pump is not shown here), leading to the injection of ψ_+-spin excitons in the reservoir. The temporal evolution of the blueshift derived from the ψ_+ and ψ_- excitons is shown in Fig. S11(c), demonstrating the ψ_+ excitons indeed relaxed into ψ_-. A resonant Gaussian source is used to excite the topological edge mode once the steady state of the exciton reservoir is reached, and a snapshot of the excited topological mode is plotted in Fig. S11(d), showing that the edge mode is propagating along the edge without being back reflected, as expected.

[1] K. Y. Dixon, T. A. Loring, and A. Cerjan, Classifying Topology in Photonic Heterostructures with Gapless Environments, Physical Review Letters **131**, 213801 (2023), 2303.17135.
[2] S. Wong, T. A. Loring, and A. Cerjan, Classifying topology in photonic crystal slabs with radiative environments (2024), 2402.10347.
[3] S. Klembt, T. H. Harder, O. A. Egorov, K. Winkler, R. Ge, M. A. Bandres, M. Emmerling, L. Worschech, T. C. Liew, M. Segev, C. Schneider, and S. Höfling, Exciton-polariton topological insulator, Nature **562**, 552 (2018), 1808.03179.
[4] O. Bleu, D. D. Solnyshkov, and G. Malpuech, Interacting quantum fluid in a polariton Chern insulator, Physical Review B **93**, 1 (2016).
[5] O. Bleu, D. D. Solnyshkov, and G. Malpuech, Photonic versus electronic quantum anomalous Hall effect, Physical Review B **95**, 1 (2017), 1701.03680.
[6] D. D. Solnyshkov, O. Bleu, and G. Malpuech, Topological optical isolator based on polariton graphene, Applied Physics Letters **112**, 10.1063/1.5018902 (2018).
[7] H. Sigurdsson, Y. S. Krivosenko, I. V. Iorsh, I. A. Shelykh, and A. V. Nalitov, Spontaneous topological transitions in a honeycomb lattice of exciton-polariton condensates due to spin bifurcations, Physical Review B **100**, 235444 (2019).

[8] L. Ferrier, E. Wertz, R. Johne, D. D. Solnyshkov, P. Senellart, I. Sagnes, A. Lemaître, G. Malpuech, and J. Bloch, Interactions in Confined Polariton Condensates, Physical Review Letters **106**, 126401 (2011).

[9] R. Banerjee, S. Mandal, and T. C. Liew, Optically induced topological spin-valley Hall effect for exciton polaritons, Physical Review B **103**, 1 (2021), 2107.11716.

[10] H. Ohadi, E. Kammann, T. C. H. Liew, K. G. Lagoudakis, A. V. Kavokin, and P. G. Lagoudakis, Spontaneous Symmetry Breaking in a Polariton and Photon Laser, Physical Review Letters **109**, 016404 (2012).
